# Competing quantum effects in heavy-atom tunnelling through conical intersections†

**DOI:** 10.1039/d3sc03706a

**Published:** 2023-09-27

**Authors:** Wei Fang, Eric R. Heller, Jeremy O. Richardson

**Affiliations:** a Department of Chemistry, Fudan University Shanghai 200438 P. R. China; b Department of Chemistry and Applied Biosciences, ETH Zürich 8093 Zürich Switzerland jeremy.richardson@phys.chem.ethz.ch

## Abstract

Thermally activated chemical reactions are typically understood in terms of overcoming potential-energy barriers. However, standard rate theories break down in the presence of a conical intersection (CI) because these processes are inherently nonadiabatic, invalidating the Born–Oppenheimer approximation. Moreover, CIs give rise to intricate nuclear quantum effects such as tunnelling and the geometric phase, which are neglected by standard trajectory-based simulations and remain largely unexplored in complex molecular systems. We present new semiclassical transition-state theories based on an extension of golden-rule instanton theory to describe nonadiabatic tunnelling through CIs and thus provide an intuitive picture for the reaction mechanism. We apply the method in conjunction with first-principles electronic-structure calculations to the electron transfer in the bis(methylene)-adamantyl cation. Our study reveals a strong competition between heavy-atom tunnelling and geometric-phase effects.

## Abstract 2

The geometric-phase effect appears in reactions which tunnel around conical intersections. Our novel instanton theories indicate that this plays an important role in certain charge-transfer reactions, even at room temperature.

## Graphical abstract

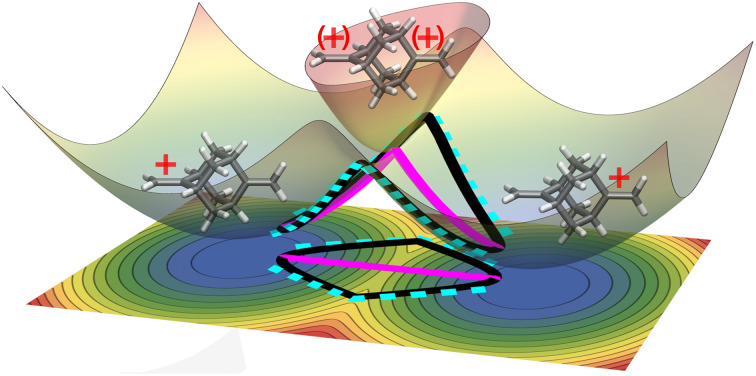

## 1 Introduction

The Born–Oppenheimer approximation is the foundation of a large part of our understanding of chemistry. However, it breaks down when a chemical process involves two (or more) electronic states that approach each other, for which nonadiabatic effects become dominant.^1–5^ Of particular interest are conical intersections (CIs), where the states touch.^6^ The presence of CIs gives rise to the ultrafast decay of excited states^7–11^ and is at the core of many biological processes vital to life.^12–14^

Although the importance of conical intersections in mediating the transition from one adiabatic state to another is now well established,^6^ CIs may also exist at the heart of thermally activated chemical reactions, in which both reactants and products are in the same adiabatic state. Nonetheless, the reaction may still be a nonadiabatic process, in which the CI plays the role of the transition state.^15^ However, whereas transition-state theory (TST) is well established and leads to simple and intuitive mechanistic interpretations in cases where the Born–Oppenheimer approximation applies, no comparable theory exists for nonadiabatic reactions involving CIs. Because of the lack of rate theories which are easy to apply and interpret, reactions involving CIs are commonly analyzed purely in terms of the electronic structure of static points along pre-determined reaction pathways. Such approaches permit rough, qualitative insight based on relative barrier heights but ignore the dynamics that emerge from the unique properties of a CI. New and rigorous approaches to compute the rate of nonadiabatic reactions mediated by CIs in the spirit of transition-state theory are thus highly desirable.

Recent studies have shown that semiclassical golden-rule instanton theory (SCI)^16–19^ can be used to unveil dramatic effects of quantum tunnelling on reaction rates and mechanisms in nonadiabatic spin-crossover reactions,^20,21^ which eluded previous theoretical analyses based on conventional (reductionist) methods.^22–24^ Surprisingly, the SCI results suggest that heavy-atom quantum tunnelling may be significant even at room temperature in nonadiabtaic reactions. The SCI method constitutes a rigorous semiclassical approximation to Fermi's golden rule (FGR) and predicts tunnelling mechanisms as well as rates based on locating optimal tunnelling pathways (called “instantons”) in full dimensionality. As the method is far more efficient than a full quantum simulation, it does not require constructing a model potential but can instead be combined with on-the-fly *ab initio* electronic-structure calculations. However, none of the previous studies included a reaction through a conical intersection. It thus remains to be answered whether quantum tunnelling may be equally important in nonadiabatic processes through conical intersections, where geometric-phase effects (GPEs)^25–30^ may also have a decisive influence on the outcome of chemical reactions.^31–43^

In this work, we extend instanton theory to describe nonadiabatic reactions through CIs in the golden-rule regime including tunnelling, zero-point energy (ZPE) and GPEs. We present a novel view on reaction mechanisms based on multiple instanton pathways that traverse, bypass or wind around the CI. By combining the extended theory with on-the-fly constrained density-functional theory (CDFT) calculations, we study charge transfer in the 2,6-bis(methylene) adamantyl (BMA) cation, a classic system featuring a CI.^44,45^ The presence of a CI creates a double-well structure in the ground adiabatic state which allows the charge to be trapped in one of the two sites for relatively long timescales, a phenomenon known as diabatic trapping. It is our goal to calculate the rate of charge transfer between the wells in thermal equilibrium.

The ground-state dynamics of the BMA cation have been the topic of a couple of previous studies. In ref. 44, a few on-the-fly surface-hopping trajectories were run at the molecule's ZPE to explore the reaction mechanism. This approach is based on classical dynamics, which has the problem of causing unphysical flow of ZPE,^46^ and additionally ignores other potentially important NQEs such as tunnelling and the GPE. In ref. 47, an implementation of FGR was applied to a linear-vibronic coupling (LVC) model. Although this method has the rigour of quantum mechanics, it sacrifices transferability by relying on a global harmonic approximation, which may or may not be valid depending on the molecule in question. In contrast, instanton theory allows us to calculate the rate in the full-dimensional molecule from first principles. Additionally, it provides mechanistic insights and allows disentangling the separate contributions from ZPE, tunnelling and geometric-phase effects. In this way, we reveal an intriguing picture of competition between heavy-atom tunnelling and significant GPEs, even at room temperature.

## 2 Results and discussion

### 2.1 Rate theory

Our nonadiabatic system is defined by the reactant (*n* = 0) and the product (*n* = 1) diabatic potential-energy surfaces (PESs), *V*_*n*_(**x**), which are functions of the nuclear coordinates, **x**. Transitions between the two states are facilitated by the diabatic coupling, Δ(**x**). In the case of weak coupling, Fermi's golden rule^48^ provides a useful definition of the reaction rate. However, even this simplified approach can only be applied to the smallest molecular systems (unless a global harmonic approximation is taken such as in the LVC model) since it requires the solution of the time-independent vibrational Schrödinger equation separately for each electronic state, which constitutes a prohibitively expensive numerical problem.

Instead of using the traditional wavefunction picture, we can utilize Feynman's path-integral formulation of quantum mechanics.^49^ Although this is formally equivalent, it is a useful starting point from which to make further approximations. In particular, we approximate the exact path-integral expression by the semiclassical van-Vleck propagators.^50^ The majority of the derivation is identical to that of our previous work^16–19^ and so we will focus only on the key differences when describing a reaction through a CI, as opposed to an avoided crossing. We start from the following expression for the SCI rate1
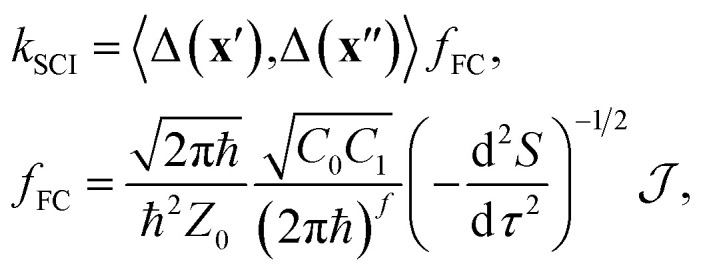
where *f*_FC_ is the semiclassical instanton approximation of the thermally weighted Franck–Condon overlaps and *Z*_0_ is the reactant partition function. The term 
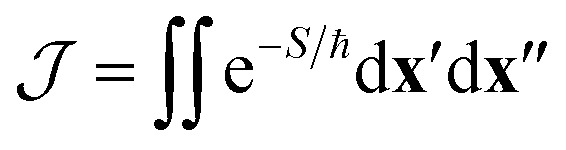
 depends on the Euclidean action, *S* = *S*_0_(**x**′, **x**′′, *β*ℏ − *τ*) + *S*_1_(**x**′′, **x**′, *τ*), given as the sum of contributions from one path travelling from **x**′ to **x**′′ in imaginary time *β*ℏ − *τ* on the reactant electronic state and another path travelling back from **x**′′ to **x**′ in imaginary time *τ* on the product electronic state, where *β* = 1/*k*_B_*T* is the inverse temperature.

The semiclassical approximation used to evaluate eqn (1) is based on locating the stationary point of *S* with respect to positions, **x**′ and **x**′′, and imaginary time, *τ*. This defines the optimal tunnelling pathway known as the “instanton.” It corresponds to a classical periodic orbit that travels below the barrier and changes electronic state at **x**′ and **x**′′, thus providing an intuitive picture of nonadiabatic quantum tunnelling.^16,18^ Typically **x**′ and **x**′′ coincide at a configuration referred to as the “hopping point.” The configuration integrals in 
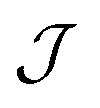
 can then be evaluated by steepest descent, which here corresponds to a semiclassical approximation that becomes exact in the ℏ → 0 limit.^51^ Because the integrand is dominated by the minimum of *S*,¶¶In the Marcus inverted regime, it is not a minimum, but a high-index saddle-point of *S* that is required.^18,19^ we can approximate it in terms of the Gaussian fluctuations of e^−*S*/ℏ^ around the stationary point. As described in ref. 16 and 18, the second derivative with respect to *τ* originates from a steepest-descent integration in time and the *C*_*n*_ are prefactors of the van-Vleck propagators. All terms in eqn (1) are evaluated at the instanton configuration.

The first term in eqn (1) is the two-point correlation2
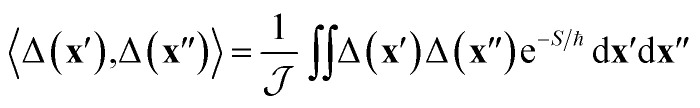
In the standard case where the reaction proceeds without a CI, the slowly-varying coupling can be set to its value at the hopping point. The rate for a reaction without CI is then given by *k*_SCI_ = Δ^2^*f*_FC_.^16–19^

When the hopping point is located on a CI seam, however, the value of the diabatic coupling is exactly zero, which invalidates the approximation as it would predict a zero rate. One therefore needs to include the next-order term in the coupling's Taylor expansion at the CI, Δ(**x**) ∼ **α·x** (assuming the origin of the coordinate system is shifted to the hopping point). This relation defines the (diabatic) coupling vector **α** = ∂Δ/∂**x**, whose direction and magnitude correspond to the coupling mode and coupling strength respectively.

The effect of the CI on the rate is therefore contained within the two-point correlation, *χ* = 〈Δ(**x**′), Δ(**x**′′)〉, which we approximate by3
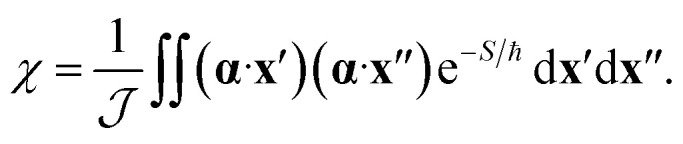
Again, we can apply a semiclassical approximation to the integrals in eqn (3) and replace the exponential by Gaussian fluctuations about the instanton. The resulting integral (a multidimensional Gaussian multiplied by a monomial) can be evaluated analytically. This approach defines the SCI method.

However, there is an alternative way of writing the integrand of eqn (2) using

4 Δ(**x**′)Δ(**x**′′)e^−*S*/ℏ^ = sgn[Δ(**x**′)Δ(**x**′′)]e^−*S*^eff^/ℏ^,

where *S*^eff^ = *S* − ℏ ln|Δ(**x**′)Δ(**x**′′)|. This effective action may exhibit several stationary points (labelled by index *a*), each giving rise to a prefactor *χ*^eff^_*a*_ = sgn[Δ(**x**′)Δ(**x**′′)], depending on the relative sign of the diabatic-coupling terms. That is, if at the stationary point **x**′ and **x**′′ are on the same side of the CI, then *χ*^eff^_*a*_ = 1, but if they are on opposite sides, then *χ*^eff^_*a*_ = −1. The total rate is given by a sum over the contributions from each stationary point5
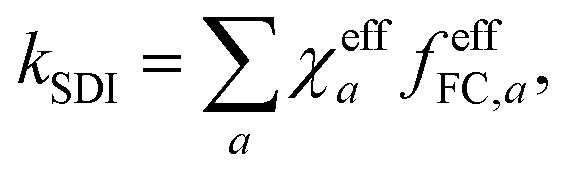
where *f*^eff^_FC,*a*_ is evaluated by steepest descent (as in eqn (1) except using *S*^eff^ in place of *S*) around the stationary point *a*. For a single CI with linear diabatic coupling, we expect four stationary points of the effective action, which we will call “steepest-descent instantons” (SDIs), two with a positive contribution to the total rate and two with a negative contribution (see ESI† for an illustration).

The SCI and SDI approaches both describe the changes to the molecular fluctuations due to the existence of a CI and therefore due to an intricate interplay of ZPE, quantum tunnelling and GPEs. In the SCI rate, the GPE information is contained in the factor *χ*. In the SDI formulation, it instead manifests itself in the occurrence of more than one instanton with positive and negative contributions to the rate.

Finally, it is also possible to formulate a classical golden-rule rate through a conical intersection by taking the high-temperature limit of the SCI rate. In this case, the instanton collapses to the minimum of the crossing seam between the diabatic potentials. If this minimum-energy crossing point (MECP) is a CI, we refer to it as the minimum-energy conical intersection (MECI). This gives the classical rate constant,6
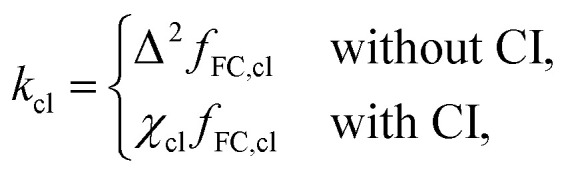
where *f*_FC,cl_ is the classical limit of the Franck–Condon factor.^16,19^ Note that this expression reduces to Marcus theory (or its extension to treat a CI)^52,53^ for a system with harmonic free-energy surfaces. Alternatively, by replacing the classical harmonic vibrational partition functions in *f*_FC,cl_ with their quantum analogue, an Eyring-like nonadiabatic transition-state theory (NA-TST)^54–57^ rate can be computed, which approximately includes ZPEs but not quantum tunnelling. For a system with a CI, *χ*_cl_ = 〈(**α**·**x**)^2^〉_cl_ is the classical limit of *χ*, with the key difference being that the integrand in eqn (3) is positive definite and hence does not capture the GPE.

### 2.2 Optimal tunnelling pathways through conical intersections

**Fig. 1:**
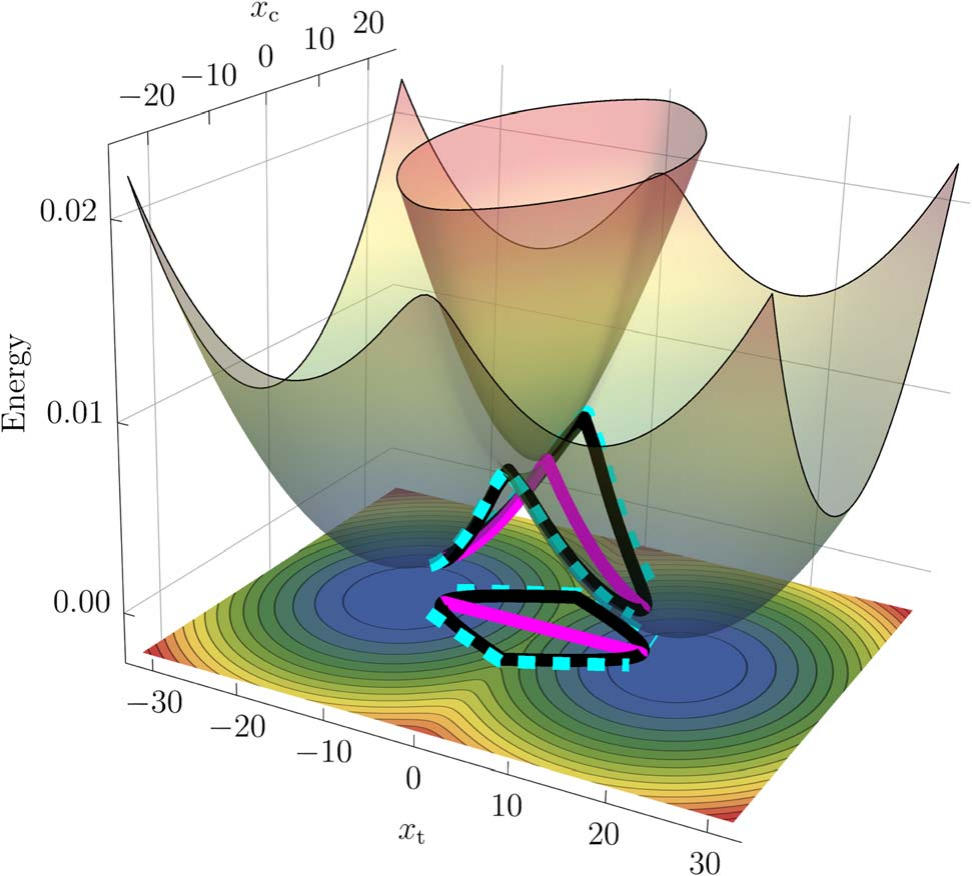
Surface plot of the adiabatic potentials in the *x*_t_*x*_c_-plane with relaxed bath mode *x*_b_. The straight SCI path (magenta) as well as the curving (cyan) and winding (black) SDI paths are illustrated on the surfaces and on the corresponding contour plot of the adiabatic ground state at the bottom.

While nonadiabatic instanton theory has been successfully applied to a number of chemical reactions, this is the first application of the theory to a process involving a conical intersection. We examine the theory's validity by first considering a benchmark reduced-dimensionality harmonic model for the BMA cation from the literature^58,59^ shown in Fig. 1. The Figure depicts the CI in the space spanned by the coupling mode, *x*_c_, which points in the direction of **α**, and the orthogonal tuning mode, *x*_t_, which points in the direction of the gradient-difference vector calculated at the CI. This is the minimal description of the *branching space*, which lifts the degeneracy of the adiabatic potentials around the CI. In a system with more than two internal degrees of freedom, the remaining modes form the crossing seam between the adiabatic potentials. We extend the two-dimensional system by adding a third mode with a low frequency to approximately account for the dissipative effect of the remaining molecular modes. The model parameters are given in the ESI.†

Due to the simplicity of the harmonic model, it is possible to compute the non-perturbative quantum-mechanical rate as well as the FGR rate from the flux correlation function^60,61^ (see ESI† for details). The results are given alongside the instanton rates in Table 1, including the Born–Oppenheimer instanton (BOI) rate.^62–67^

**Table 1:** Reaction rates [in ns^−1^] at 300 K for the three-mode model computed with the SCI, its resummed version (rSCI, see ESI for details) and SDI methods as well as with the quantum-mechanical FGR and non-perturbative quantum mechanics (exact). For comparison, we also give the Born–Oppenheimer instanton (BOI) rate

BOI	SCI	SDI	rSCI	FGR	Exact
2401	3.306	1.784	1.507	1.473	1.475

This simple model is not expected to faithfully describe the full-dimensional molecule. However, it allows us to compare the performance of the different methods.

The excellent agreement between the non-perturbative and FGR results confirms that the charge-transfer process in the BMA cation is in the GR regime. This conclusion is in agreement with the results of ref. 47 from dynamics simulations on a higher-dimensional LVC model of the BMA cation, which is discussed further in the ESI.† Correspondingly, the Born–Oppenheimer approximation breaks down and BOI overestimates the rate by more than 3 orders of magnitude. It is therefore clear that the reaction in the BMA cation is a fundamentally nonadiabatic process, which cannot be described by dynamics on the adiabatic ground state.

The SDI method gives a good estimate of the FGR rate. The SCI result still captures the correct order of magnitude, but deviates from the exact result by about a factor of 2. This error is caused by the steepest-descent integration over time that we took to arrive at eqn (1). In the ESI,† we derive a resummed SCI theory (rSCI) for the harmonic model that (approximately) corrects for this effect by including higher-order derivatives of *χ*. It can be seen from Table 1 that this approach leads to excellent agreement with the quantum-mechanical results. We note, however, that the resummation requires further generalizations to be applicable to more general systems. Nonetheless, even without this correction, the SCI result is well within the typical error margins of *ab initio* studies.

In addition to the calculation of accurate rates, instanton theory provides direct insight into the reaction mechanism by locating the optimal tunnelling pathways. We present the instantons in the SCI and SDI formalisms on a 2D surface plot of the model adiabatic PESs in Fig. 1.

It can be seen that the SCI pathway (magenta) goes straight through the conical intersection, whereas there are two direct SDI pathways (cyan) that curve around the left and right sides of the CI as well as a pair of equivalent paths (black) that wind around it in a clockwise and counterclockwise direction. Note that the total SDI rate presented in Table 1 is the sum of two contributions; while the curving paths give rise to a positive rate of 21.40 ns^−1^, the winding instantons give a negative contribution of −19.61 ns^−1^. The latter do not have a classical analogue (see ESI† for more details) and are a manifestation of the GPE. The winding paths therefore provide an intriguing picture of how the GPE reduces the reaction rate, reminiscent of the topological formalism developed in ref. 32, 33 and 37.

Within the SCI method, the GPE is also included by accounting for fluctuations into the four quadrants of the integral in eqn (3). Fluctuations into two of the quadrants give paths which do not wind around the CI and thus result in positive contributions, whereas fluctuations in the other two quadrants give paths which wind around the CI and result in negative contributions (see Fig. SI.2 of the ESI†). In this way, the SCI approach is able to account for the GPE from four different quadrants using only one instanton. In addition, the SCI approach only requires a calculation of the diabatic coupling once after the optimization is complete, rendering it more efficient than the SDI method. In the following, we will therefore base our *ab initio* analysis of the BMA cation on the SCI approach.

### 2.3 First-principles study

**Fig. 2:**
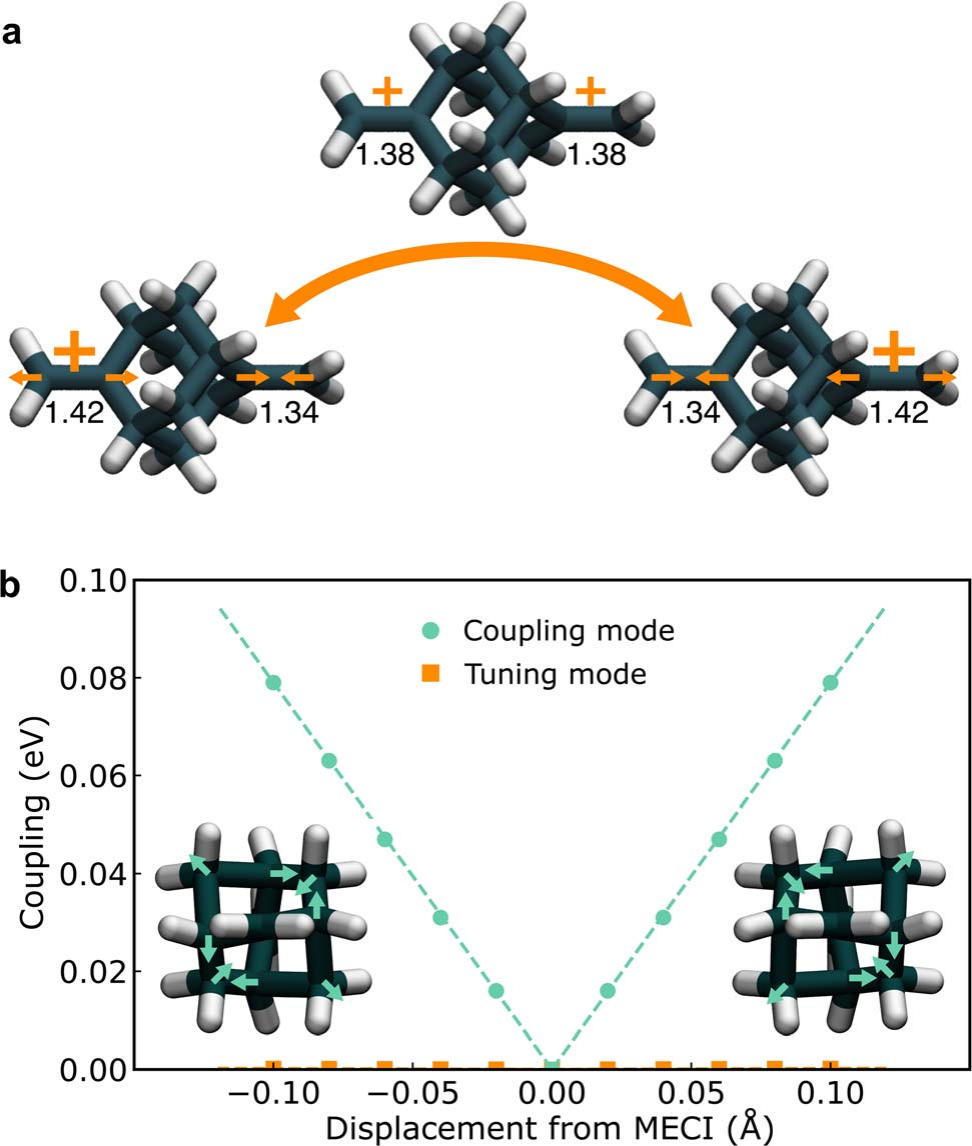
(a) Reactant, MECI, and product geometries for the charge transfer in the 2,6-bis(methylene) adamantyl (BMA) cation. The C=C bond lengths are also shown (in Å). The arrows indicate the predominant movements of carbon atoms in the tuning mode. (b) Absolute value of the diabatic electronic coupling, |Δ|, along the coupling and tuning modes. The dashed lines are linear fits. The insets illustrate the distortion of the molecule along the coupling mode and the arrows show the predominant movements of carbon atoms.

To locate the key configurations in the electron-transfer reaction of the BMA cation, we employed constrained density functional theory (CDFT),^68^ which naturally provides potentials in the diabatic representation, and performed a full-dimensional optimization of the minimum and MECP (illustrated in Fig. 2a). The MECP has *D*_2d_ point-group symmetry while the reactant and product minimum geometries have *C*_2v_ symmetry. The computed MECP barrier is *V*^‡^ = 0.171 eV, in agreement with a previous study using CASSCF, which reported the barrier to be in the range 0.142–0.197 eV.^44^ The coupling at the MECP was found to be zero, implying that the MECP is located on the CI seam, and is thus identified with the MECI.

At the CI, as introduced in Section 2.2, there are two important modes that lift the degeneracy between the adiabatic states, namely the tuning mode and the coupling mode.^69^ For BMA, the tuning mode mainly consists of the contraction of one C=C bond and the elongation of the other one (Fig. 2a). The onset temperature^21^ (an analogue to the crossover temperature for adiabatic reactions) is 505 K, indicating that significant tunnelling effects are expected at temperatures below this value. The coupling mode can also be identified using CDFT (see ESI† for details), which is a symmetry-breaking distortion of the cage structure of BMA (Fig. 2b), previously described as “asymmetric breathing” in ref. 44, with a frequency of 1060 cm^−1^. Displacing along the coupling mode lowers the symmetry, and while the diabatic states remain degenerate, Δ increases linearly for small displacements, as shown in Fig. 2b. The slope is ‖**α**‖ = 0.79 eV Å^−1^. Although we used a completely different theory to compute **α** compared to previous studies in the literature,^44,58^ the results are fairly close (within a factor of two), indicating again that CDFT models the two diabatic states in this system reasonably well.

**Fig. 3:**
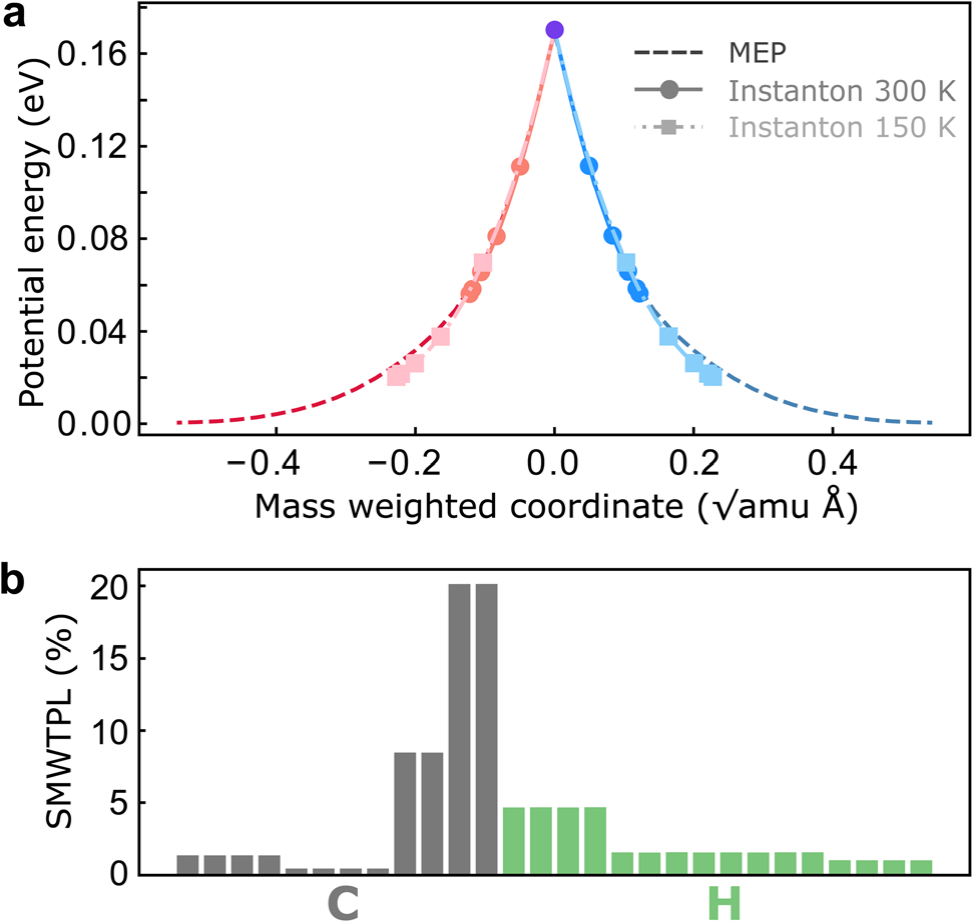
Analysis of the instanton trajectories. (a) Potential-energy profile along the instanton pathways at 300 K and 150 K compared with the minimum-energy pathway (MEP). The red (blue) beads are associated with the reactant (product) diabatic surface. The violet bead marks the hopping point. (b) Squared mass-weighted tunnelling path length (SMWTPL) computed from the instanton trajectory at 300 K for each atom as a percentage of the total SMWTPL.

To investigate the role of NQEs in charge transfer through the CI in BMA, we performed SCI optimizations over a range of temperatures from 370 K to 150 K. Before discussing the computed rates, we report the key features and atomistic details of the tunnelling mechanism offered by instanton theory. In all cases, the hopping point of the instanton is located at the MECI due to symmetry. As expected from our analysis above, instanton trajectories reveal that even at room temperature, the instanton is delocalized along the reaction coordinate (Fig. 3a), which is an indication of significant tunnelling effects in this system. Many atoms are involved in the tunnelling process, and surprisingly, the tunnelling of the heavy C atoms is as important as the tunnelling of the light H atoms (Fig. 3b). It is worth pointing out that predictions of heavy-atom tunnelling at room temperature have been scarce,^20,21^ as it is typically considered to be only viable at very low temperatures.^70–73^ We attribute this to the shape of the underlying PESs in nonadiabatic reactions (Fig. 3a), which feature a sharp cusp along which the instanton can stretch. No significant corner-cutting effect has been observed as the instanton trajectories almost lie on top of the minimum-energy pathway (Fig. 3a). One might therefore assume that a simple tunnelling correction based on this one-dimensional potential profile would be sufficient. However, we shall show that there are important multidimensional contributions from the fluctuations perpendicular to the pathway, which are captured by the full-dimensional instanton theory. Another key advantage of instanton theory is that the mechanism is clearly revealed and the relative contributions to the rate can be analyzed separately. This is the subject of the next subsection.

### 2.4 Competing quantum effects

**Fig. 4:**
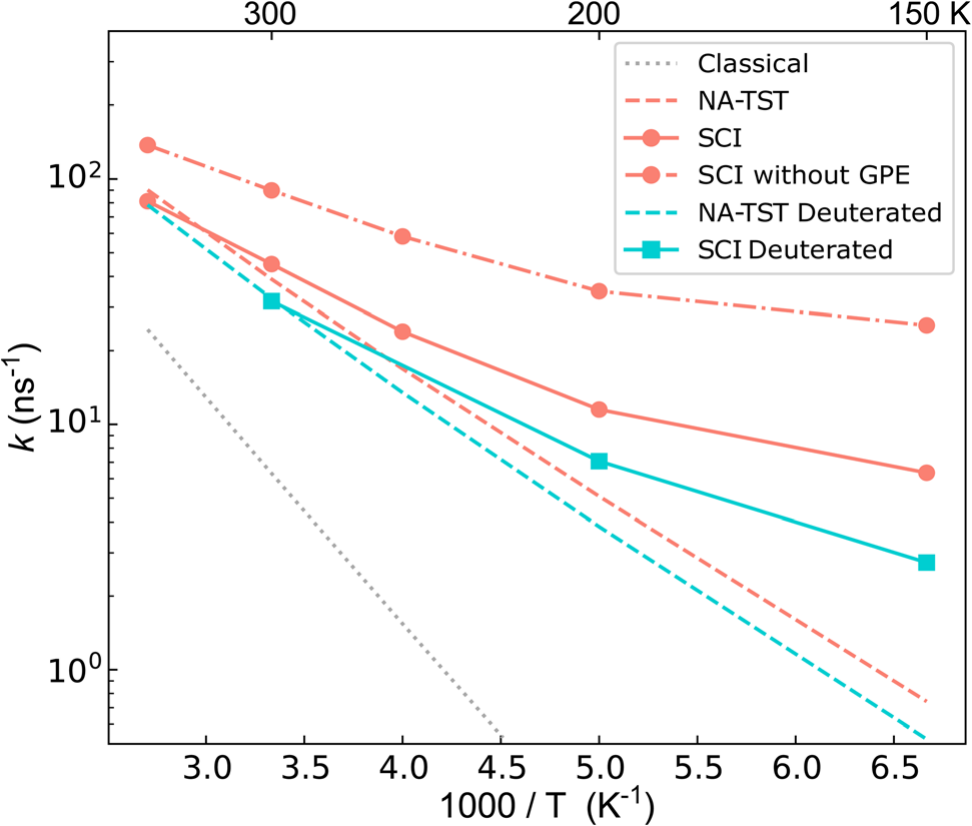
Semiclassical instanton (SCI) rate constants computed for the charge transfer process in the BMA cation. The classical limit of SCI, the (ZPE corrected) NA-TST, and the SCI excluding GPE (*i.e.* with *χ*_cl_ instead of *χ*) are also plotted for comparison. The turquoise squares show the SCI result computed for the fully deuterated BMA cation.

The calculated rates are shown in Fig. 4. First, we compare the NA-TST rate,^54–57^ which incorporates harmonic ZPE *via* a simple Eyring-like correction, with *k*_cl_. It is clear that there are large ZPE effects, which result from an effective lowering of the MECI barrier by 0.085 eV. Next, we compare the instanton (SCI) rates with NA-TST. At room temperature, the SCI rate is only marginally higher than the NA-TST rate, and even at 150 K, it is only one order of magnitude higher. This seems to imply that, at least at room temperature, the mechanism can be well understood by simply accounting for ZPE within the otherwise classical mechanism of NA-TST. However, as we shall show, this is not the full story.

One can straightforwardly decompose the instanton rate into different contributions, providing insights into how different NQEs impact the charge transfer through a CI. The various contributions are given in Table 2 for a range of temperatures. As already explained, there is a significant ZPE correction (defined as the ratio of the NA-TST rate to the classical rate).

**Table 2:** Decomposition of the SCI rate constant into contributions from different NQEs. The contribution from ZPE is given by the ratio *k*_NA-TST_/*k*_cl_ and from quantum tunnelling by e^−*S*/ℏ^/e^−*βV*^‡^^. CI effects, *i.e.* non-separable multidimensional NQEs induced by the CI including GPEs, is given by the ratio *χ*/*χ*_cl_. The contribution from fluctuations is defined as the ratio between SCI and NA-TST prefactors. The product of the four terms gives the total quantum correction factor *k*_SCI_/*k*_cl_

*T* [K]	ZPE	Tunnelling	CI	Fluctuations
370	3.7	4.0	0.59	0.38
300	6.2	9.1	0.50	0.26
250	11	22	0.41	0.15
200	27	100	0.33	0.069
150	120	1490	0.25	0.023

The contribution to the rate from tunnelling is defined as the ratio between the exponential part of the instanton rate (e^−*S*/ℏ^) and that of the classical rate (e^−*βV*^‡^^). This reports solely on the probability of barrier penetration *vs.* thermal activation along the reaction coordinate.^62^ It is seen that even at room temperature, heavy-atom tunnelling accelerates the rate of charge transfer through the CI by almost an order of magnitude. Tunnelling contributions increase dramatically as the temperature decreases; at 150 K, it can speed up charge transfer by three orders of magnitude. These significant tunnelling contributions point towards a story that “non-trivial” NQEs (*i.e.* not just ZPE) can drastically speed up charge transfer through a CI. This is, however, in apparent conflict with the rates in Fig. 4.

To understand the counter-intuitive findings, we examine the other contributions in the SCI rate. The two-point correlation, *χ*, encapsulates multidimensional NQEs induced by the CI, including the GPE. This effect reduces the rate at room temperature by a factor of 2. As temperature decreases, the rate reduction induced by GPE increases to a factor of 4 at 150 K. Thus, if one excludes the GPE by using *χ*_cl_ instead of *χ*, the instanton rate becomes substantially faster than the NA-TST rate, which is the expected behaviour for a reaction with significant tunnelling. As was seen in the model system, the SCI approximation overestimates the rate. We thus expect the true GPE contribution to be even more substantial than implied by Table 2.

Finally, instanton theory captures multidimensional fluctuation effects in the prefactor. The fluctuations parallel to the pathway capture the quantum analogue of the Landau–Zener nonadiabatic transmission coefficient, whereas the fluctuations perpendicular to the pathway encode information about ZPE change along the tunnelling pathway. Table 2 shows that fluctuations reduce the rate, and in the ESI,† we show that this is a multidimensional effect as it becomes insignificant if computed on the 1D MEP. This also suggests that despite no obvious corner-cutting effect (*i.e.* the action, *S*, computed on the 1D MEP is within 2% of that computed with multidimensional instanton theory), one should be cautious of relying on 1D tunnelling methods.

Overall, the above results demonstrate that closely competing quantum effects exist, specifically significant heavy-atom tunnelling which increases the rate, countered by strong GPEs as well as multidimensional fluctuation effects, which act to reduce the rate. This explains why the total effect from non-trivial NQEs appears small.

We can provide further justification for our conclusions based on a study of the kinetic isotope effect (KIE). KIEs are often used as an unambiguous observable for probing the role of NQEs in chemical reactions. By performing SCI calculations on fully deuterated BMA, we found H/D KIEs of 1.4 at room temperature, which increases to 2.3 at 150 K. Such large factors cannot be explained simply in terms of ZPE corrections (see ESI†). This points at the existence of non-trivial NQEs even though the SCI rate is almost equal to the NA-TST rate. At first sight, it may seem surprising that deuteration has such a minor effect, which appears to contradict our statement that tunnelling is so significant. However, it is reconciled by the fact that there is considerable contribution to the tunnelling from the heavy C atoms, which is of course unaffected by the deuteration. Indeed, we estimate the contribution from the tunnelling factor to the ^12^C/^13^C KIE to be 1.06 at room temperature, which is remarkably large considering the relatively small change in the mass.

## 3 Conclusions

Before concluding, we discuss the necessity of performing a full-dimensional *ab initio* simulation. While the *ab initio* SCI rate at room temperature is similar to the SCI rate computed on the LVC model constructed by Izmaylov *et al.*,^47^ the temperature dependence of the two rates differs qualitatively (see ESI†). Specifically, the LVC rates become almost temperature independent below 300 K while the *ab initio* rates decrease with temperature, showing a non-Arrhenius behaviour. Further analysis (as detailed in the ESI†) shows that the difference comes predominately from tunnelling, suggesting that the LVC model is inadequate for accurately describing the barrier shape along the instanton. This finding is also similar to what we have shown in previous works, *i.e.* qualitative improvements in using instanton theory over global harmonic models.^20,21^ While it would be possible to re-parameterize the LVC model to achieve better agreement with the CDFT results for BMA, the globally harmonic form of the model will still remain a major limiting factor.

In this work, we extended instanton theory to compute quantum tunnelling rates through CIs in the golden-rule regime, where the diabatic electronic coupling changes slowly in moving away from the CI. While this is valid for the BMA cation, in more complicated systems where the coupling is stronger, instanton theory could be extended beyond the golden-rule limit following ref. 74. Additionally, the theory can be straightforwardly generalized for sloped CIs (corresponding to the Marcus inverted regime) in the same way as in ref. 18. Hence, instanton theory constitutes a powerful method for modelling and understanding NQEs in reactions through CIs. It automatically accounts for the geometric-phase effect by assigning negative weights to paths which wind around the CI.

In summary, combining instanton theory and on-the-fly CDFT calculations, we investigated NQEs for the charge transfer process in the BMA cation, a well-known system for diabatic trapping. Even at room temperature, our analysis reveals the competition of different quantum effects, specifically multidimensional NQEs induced by the conical intersection such as the geometric-phase effect hinder the charge-transfer process and cancel out part of the speed-up gained from heavy-atom tunnelling.

## 4 Methods

### 4.1 Electronic structure

We employ CDFT, a widely applied method for computing diabatic states and their couplings, which provides a good balance between accuracy and computational cost.^4,68,75^ The reactant (product) diabatic PES has the positive charge constrained on the left (right) side of the molecule (see Fig. 2a). The constraint condition is the difference between the number of electrons (±1) on the left arm (which includes the two C atoms in the double bond as well as the 2H and 2C atoms connected to them) and the right arm of the molecule. CDFT enforces the constraint *via* a Lagrange multiplier, and we use a tight constraint convergence condition of 0.01 electrons in the CDFT iterations. The CDFT calculations are performed using the CP2K program package.^76–78^ The PBE functional^79^ is used in the instanton optimizations, and the hybrid PBEX50 functional (PBE with 50% Fock exchange) is used for computing diabatic couplings. A plane-wave cutoff of 280 Ry (320 Ry) is used for the PBE (PBEX50) functional. We have checked that the energy and diabatic electronic coupling are converged with respect to the plane-wave cutoff. The unit cell size is 20 Å ×20 Å ×20 Å. The molecular optimized GTH basis set is used for the PBE part, and the ADMM method with the pFIT3 (ref. 80) basis set is used for computing Fock exchange. The PBE and PBEX50 functional predict almost the same reactant and MECI geometries, MECI barrier and reorganization energy for the BMA cation (see ESI†).

### 4.2 Instanton theory

Instantons are located using a ring-polymer optimization^17^ with on-the-fly calls to CDFT until the total force converged to below 0.03 eV Å^−1^. *N* = 20 beads are used to represent the full ring-polymer instanton path (except at 370 K where *N* = 12 beads were used). However, we take advantage of the fact that only *N*/2 + 1 of the beads are independent, due to the fact that the ring polymer folds back upon itself.^19,63^ Since the charge-transfer process is symmetric, the beads were split evenly between the reactant and product, with *τ* = *β*ℏ/2. The instanton rate is extrapolated to the infinite bead limit by multiplying by the factor *k*_NA-TST_(∞)/*k*_NA-TST_(*N*), using a similar approach as has been used for adiabatic instantons.^81^ Note that this correction only has a minor effect on the rates here. We also optimized the instanton with 32 beads at the lowest two temperatures (200 and 150 K) and found that the SCI rates change by less than 10% going from 20 to 32 beads. The computational bottleneck is typically the 12 numerical hessian calculations performed after the optimization (each of which takes about 500 CPU hours). We note that this is trivially parallelized and could be further sped up using interpolation methods.^82^

## Data availability

The data supporting the findings of this study are available within the paper and its ESI† files.

## Author contributions

E. R. H. and J. O. R. developed the theory for the model system, W. F. implemented and ran the calculations for the *ab initio* system. All authors analysed the data and wrote the manuscript.

## Conflicts of interest

There are no conflicts to declare.

## Supplementary Material

SC-014-D3SC03706A-s001

SC-014-D3SC03706A-s002
