# Supplementary material for: Competing quantum effects in heavy-atom tunnelling through conical intersections
Source: Chem Sci. 2023 Sep 27;14(39):10777–85. doi: 10.1039/d3sc03706a (PMC10566476; doi:10.1039/d3sc03706a)
Supplement: SC-014-D3SC03706A-s002 [file SC-014-D3SC03706A-s002.pdf]

Electronic Supplementary Material (ESI) for Chemical Science.
This journal is © The Royal Society of Chemistry 2023

# Supplementary Information: Competing quantum effects in heavy-atom tunnelling through conical intersections

Wei Fang,[†,‡,¶] Eric R. Heller,[‡,¶] and Jeremy O. Richardson[*,‡]

†*Department of Chemistry, Fudan University, Shanghai 200438, P. R. China*

‡*Department of Chemistry and Applied Biosciences, ETH Zürich, 8093 Zürich, Switzerland*

¶*Contributed equally to this work*

E-mail: jeremy.richardson@phys.chem.ethz.ch

## SI.1 Model system

The motivation for complementing our *ab initio* study of the BMA cation with a related model system is to illustrate how instanton theory compares to exact quantum-mechanical results. We can thus show the adequacy of our method, which includes both the GR approximation and the semiclassical/steepest-descent approximations. It also allows us to elaborate on the instanton picture for a reaction through a conical intersection. We do not, however, expect quantitative agreement between the model system and the full-dimensional first-principles study.

Due to the general interest in the mechanism of diabatic trapping in the BMA cation, there exists a two-dimensional model potential for the molecule in the literature.[1,2] As is common for minimal models of conical intersections, the potentials are defined in terms of the coupling mode, $\hat{x}_\mathrm{c}$, and the orthogonal tuning mode, $\hat{x}_\mathrm{t}$, which together span the

Table SI.1: Parameters of the three-mode model.

| | |
|---|---|
| $T$ [K] | 300 |
| $\omega_{\mathrm{t/c/b}}$ [$\mathrm{cm}^{-1}$] | 1699, 1466, 500 |
| $\sqrt{m}\zeta_{\mathrm{t}}$ [$\sqrt{\mathrm{u}}$Å] | 0.192 |
| $c_{\mathrm{b}}/m\omega_{\mathrm{b}}^2$ | 3.6951 |
| $\alpha/\sqrt{m}$ [eV/$\sqrt{\mathrm{u}}$Å] | 0.178 |

branching space. We add an additional mode, $\hat{x}_{\mathrm{b}}$, that couples to the tuning mode with coupling strength, $c_{\mathrm{b}}$, and approximately accounts for the frictional effect of the remaining molecular modes. The potentials as functions of these three modes are defined as

$$V_n = \tfrac{1}{2}m\omega_{\mathrm{t}}^2(\hat{x}_{\mathrm{t}} \pm \zeta_{\mathrm{t}})^2 + \tfrac{1}{2}m\omega_{\mathrm{c}}^2\hat{x}_{\mathrm{c}}^2 + \tfrac{1}{2}m\omega_{\mathrm{b}}^2\left(\hat{x}_{\mathrm{b}} - \frac{c_{\mathrm{b}}}{m\omega_{\mathrm{b}}^2}\hat{x}_{\mathrm{t}}\right)^2, \tag{SI.1}$$

where the positive (negative) sign corresponds to the reactant (product) surface and the parameters are defined in Table SI.1. The parameters are mass-weighted such that $m$ is arbitrary and can thus be chosen as $m = 1$. The diabatic coupling is $\Delta(\boldsymbol{x}) = \boldsymbol{\alpha}\cdot\boldsymbol{x} = \alpha x_{\mathrm{c}}$, where $\alpha = \|\boldsymbol{\alpha}\|$.

## SI.2 Rate theory

The diabatic Hamiltonian for a general nonadiabatic reaction is given by

$$\hat{H} = \hat{H}_0\,|0\rangle\langle 0| + \hat{H}_1\,|1\rangle\langle 1| + \Delta\big(\,|0\rangle\langle 1| + |1\rangle\langle 0|\,\big). \tag{SI.2}$$

The nuclear Hamiltonian of electronic state $n \in \{0,1\}$ is of the form

$$\hat{H}_n = \sum_{j=1}^{f}\frac{\hat{p}_j^2}{2m} + V_n(\hat{\boldsymbol{x}}), \tag{SI.3}$$

where $\hat{\boldsymbol{x}} = (\hat{x}_{\mathrm{t}}, \hat{x}_{\mathrm{c}}, \hat{x}_3, \dots, \hat{x}_f)$ is the coordinate vector comprising the tuning mode ($\hat{x}_{\mathrm{t}}$), the coupling mode ($\hat{x}_{\mathrm{c}}$) as well as $f-2$ further nuclear degrees of freedom. Without loss of

generality, the nuclear degrees of freedom have been mass-weighted such that each has the same mass, $m$. The nuclei move on the potentials $V_n(\hat{\boldsymbol{x}})$ with conjugate momenta $\hat{p}_j$.

### SI.2.1 Quantum-mechanical and classical rates

The full quantum-mechanical thermal reaction rate constant is given by[3–5]

$$k\,Z_0 = \int_{-\infty}^{\infty} c(\tau + \mathrm{i}t)\,\mathrm{d}t, \tag{SI.4}$$

in terms of the reactant partition function $Z_0 = \mathrm{Tr}\left\{\mathrm{e}^{-\beta \hat{H}}\,|0\rangle\langle 0|\right\}$ and the flux correlation function

$$c(\tau + \mathrm{i}t) = \frac{1}{2}\,\mathrm{Tr}\left\{\mathrm{e}^{-\hat{H}(\beta\hbar-\tau)/\hbar}\,\hat{F}\,\mathrm{e}^{-\hat{H}\tau/\hbar}\,\hat{F}(t)\right\}. \tag{SI.5}$$

Here, $\hat{F}(t) = \mathrm{e}^{\mathrm{i}\hat{H}t/\hbar}\,\hat{F}\,\mathrm{e}^{-\mathrm{i}\hat{H}t/\hbar}$ describes the time evolution of the flux operator $\hat{F} = \frac{\mathrm{i}}{\hbar}\big[\hat{H}, |1\rangle\langle 1|\,\big]$, which for our Hamiltonian evaluates to $\hat{F} = \frac{\mathrm{i}\Delta(\hat{\boldsymbol{x}})}{\hbar}\big(\,|0\rangle\langle 1| - |1\rangle\langle 0|\,\big)$, and $\tau$ is an imaginary time which can be chosen arbitrarily (at least within the range $[0, \beta\hbar]$) without changing the result.

For the simple harmonic model [Eq. (SI.1)], the exact rate can be computed by expanding the Hamiltonian and flux operators in a basis of vibronic states. The linearly coupled system–bath model in Eq. (SI.1) can be transformed to a normal-mode basis which diagonalizes the potential energy. In the basis of normal modes, $\{\hat{q}_i\}$, with corresponding frequencies $\{\Omega_i\}$ and shifts $\{\xi_i\}$, the potentials are given by

$$V_n = \sum_{i=1}^{f} \frac{1}{2} m \Omega_i^2 (\hat{q}_i \pm \xi_i)^2, \tag{SI.6}$$

where again the positive (negative) sign corresponds to the reactant (product) surface. Note that because the coupling mode of the system–bath model is not coupled to the other modes, it will not be altered by the normal-mode transform and thus $\omega_{\mathrm{c}}$ appears in the set $\{\Omega_i\}$.

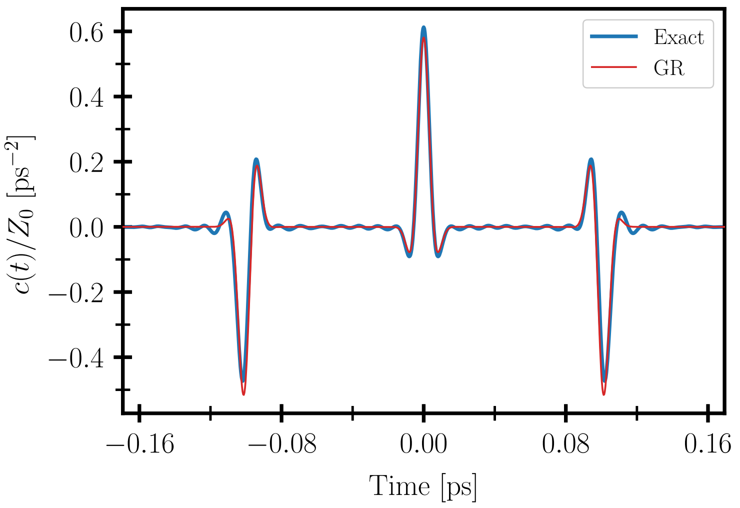


Figure SI.1: Full (exact) quantum-mechanical and golden-rule (GR) flux correlation functions as a function of time. For both correlation functions, we choose $\tau = \beta\hbar/2$. To obtain a well-defined rate constant that can be compared to the instanton results, we integrate the correlation function numerically over the interval $[-t_\mathrm{p}, t_\mathrm{p}]$, which encompasses the central peak only. Here we choose a plateau time of $t_\mathrm{p} = 0.05\,\mathrm{ps}$. We thus ignore resonances at later times, which we assume will be damped out by friction from the other modes in the full-dimensional system. For the calculation of the full quantum-mechanical correlation function, we employed a basis of $9 \times 15 \times 30$ vibrational wavefunctions for the tuning, coupling and bath modes on each electronic state.

Since the three normal modes are uncoupled, the vibrational eigenstates $(\psi_0^\mu, \psi_1^\nu)$ of the nuclear Hamiltonians, $\hat{H}_n$, with corresponding energies $(E_0^\mu, E_1^\nu)$ can be constructed as direct products of the eigenstates of the individual modes. The vibronic states are formed as a direct product of the vibrational states with electronic states $|n\rangle$.

We then evaluate the matrix exponentials in Eq. (SI.4) in a truncated vibronic basis, enabling the calculation of the flux correlation function. The result needs to be converged with respect to the size of the vibronic basis. The final time integration over the correlation function is carried out numerically. The integration limits are reduced to $[-t_\mathrm{p}, t_\mathrm{p}]$ so as to encompass the first plateau only, as illustrated in Fig. SI.1. This permits the definition of a rate in a finite-dimensional bound system.

#### SI.2.1.1 Fermi's golden rule

We now consider the golden-rule approximation. For small coupling strength, $\Delta(\hat{\boldsymbol{x}})$, Eq. (SI.4) can be expanded in a perturbation series and approximated by the first-order term, which results in the following expression for the golden-rule rate constant[6,7]

$$\begin{aligned} k_{\mathrm{GR}}\, Z_0 &= \int_{-\infty}^{\infty} c_{\mathrm{GR}}(\tau + \mathrm{i}t)\, \mathrm{d}t, \\ c_{\mathrm{GR}}(\tau + \mathrm{i}t) &= \frac{1}{\hbar^2} \operatorname{tr}\left\{ \Delta(\hat{\boldsymbol{x}})\, \mathrm{e}^{-\hat{H}_0(\beta\hbar - \tau - \mathrm{i}t)/\hbar}\, \Delta(\hat{\boldsymbol{x}})\, \mathrm{e}^{-\hat{H}_1(\tau + \mathrm{i}t)/\hbar} \right\}, \end{aligned} \tag{SI.7}$$

with the golden-rule flux correlation function, $c_{\mathrm{GR}}(\tau + \mathrm{i}t)$. The trace, $\operatorname{tr}\{\cdot\}$, is over nuclear degrees of freedom only. To arrive at the famous Fermi's golden-rule rate[8,9] generalized for an initial thermal distribution,[10,11] one evaluates the trace in the basis of reactant and product vibrational eigenstates and takes the time integral analytically

$$k_{\mathrm{GR}}\, Z_0 = \frac{2\pi}{\hbar} \sum_{\mu} \mathrm{e}^{-\beta E_0^{\mu}} \sum_{\nu} \left| \langle \psi_0^{\mu} \,|\, \Delta(\hat{\boldsymbol{x}}) \,|\, \psi_1^{\nu} \rangle \right|^2 \delta_{\mathrm{s}}(E_0^{\mu} - E_1^{\nu}). \tag{SI.8}$$

In order to define a rate in the bound three-mode model defined above, one can employ the function

$$\delta_{\mathrm{s}}(E_0^{\mu} - E_1^{\nu}) = \int_{-t_{\mathrm{p}}}^{t_{\mathrm{p}}} \frac{\mathrm{e}^{-\mathrm{i}(E_0^{\mu} - E_1^{\nu})t/\hbar}}{2\pi\hbar}\, \mathrm{d}t = \frac{\sin[(E_0^{\mu} - E_1^{\nu})t_{\mathrm{p}}/\hbar]}{\pi(E_0^{\mu} - E_1^{\nu})}, \tag{SI.9}$$

which effectively smears the harmonic-oscillator energy levels.

#### SI.2.1.2 Path-integral formulation of Fermi's golden rule

Instead of using the standard Fermi's golden rule, we wish to find an expression for the rate that does not require knowledge of the vibrational wavefunctions. Inserting the linear form

of the diabatic coupling at a CI into the correlation function in Eq. (SI.7) leads to

$$c_{\mathrm{GR}}(\tau + \mathrm{i}t) = \frac{1}{\hbar^2} \,\mathrm{tr}\left\{ (\boldsymbol{\alpha} \cdot \hat{\boldsymbol{x}})\, \mathrm{e}^{-\hat{H}_0(\beta\hbar - \tau - \mathrm{i}t)/\hbar}\, (\boldsymbol{\alpha} \cdot \hat{\boldsymbol{x}})\, \mathrm{e}^{-\hat{H}_1(\tau + \mathrm{i}t)/\hbar} \right\}. \tag{SI.10}$$

The quantum golden-rule rate for our harmonic model system with linear diabatic coupling can equivalently be evaluated using path-integral theory. The expression for the path-integral propagators for a system with the potentials in Eq. (SI.6) are known[12,13] and the position integrals with monomial prefactors in Eq. (SI.10) can be solved analytically, leading to the rate expression

$$k_{\mathrm{GR}} = \frac{1}{\hbar^2} \int \chi(\tau + \mathrm{i}t)\, \mathrm{e}^{-\phi(\tau + \mathrm{i}t)/\hbar}\, \mathrm{d}t, \tag{SI.11}$$

where

$$\phi(\tau) = -\varepsilon\tau + \sum_{i=1}^{f} 2m\Omega_i \xi_i^2 \left[ \frac{1 - \cosh \Omega_i \tau}{\tanh \beta\hbar\Omega_i/2} + \sinh \Omega_i \tau \right], \tag{SI.12a}$$

$$\chi(\tau) = \frac{\hbar\alpha^2}{2m\omega_{\mathrm{c}}} \frac{\cosh \omega_{\mathrm{c}}(\beta\hbar/2 - \tau)}{\sinh \beta\hbar\omega_{\mathrm{c}}/2}. \tag{SI.12b}$$

For the charge transfer in the BMA cation, the thermodynamic driving force, $\varepsilon$, is zero and the value of $\tau$ that makes $\phi(\tau)$ stationary is $\tau = \beta\hbar/2$ by symmetry. However, we have presented the equations such that they are valid also for systems where $\varepsilon$ is non-zero.

We illustrate the GR flux correlation function in Fig. SI.1, which is seen to be in excellent agreement with the non-perturbative correlation function. In order to obtain a well-defined rate constant, we again integrate over the interval $[-t_{\mathrm{p}}, t_{\mathrm{p}}]$.

#### SI.2.1.3 Classical golden rule

The classical golden-rule rate can be computed by evaluating the trace in Eq. (SI.10) by a phase-space integral

$$k_{\mathrm{cl}} = \frac{1}{\hbar^2}\frac{1}{(2\pi\hbar)^f}\iiint_{-\infty}^{\infty} (\boldsymbol{\alpha}\cdot\boldsymbol{x})^2\, \mathrm{e}^{-\beta\|\boldsymbol{p}\|^2/2m}\, \mathrm{e}^{-\beta V_0-(V_1-V_0)(\tau+\mathrm{i}t)/\hbar}\,\mathrm{d}t\,\mathrm{d}\boldsymbol{p}\,\mathrm{d}\boldsymbol{x}, \tag{SI.13}$$

where $\boldsymbol{p}$ is the vector of momenta conjugate to the coordinates $\boldsymbol{x}$. The Gaussian integrals over the momenta are easily solved analytically and the integral over time results in a $\delta$-function. In general, the position integral cannot be solved analytically and is thus approximated by the method of steepest descent. Thereby, one locates the stationary point of the effective potential $\widetilde{V} = (1-\lambda)V_0 + \lambda V_1$ in the combined space of positions and the Lagrange multiplier $\lambda = \tau/\beta\hbar$.[14] The stationary point of $\widetilde{V}$ corresponds to the minimum-energy crossing point (MECP) of the two potentials or, in our case, the minimum-energy conical intersection (MECI). It can be viewed as the golden-rule analogue to a transition state in adiabatic reactions. Carrying out the position integrals leads to the following expression for the classical golden-rule rate[13,15]

$$\begin{aligned} k_{\mathrm{cl}} &= \chi_{\mathrm{cl}}\, f_{\mathrm{FC,cl}}, \\ f_{\mathrm{FC,cl}} &= \sqrt{\frac{2\pi m}{\beta}}\,\frac{1}{\hbar^2}\,\frac{1}{\|\boldsymbol{G}_0-\boldsymbol{G}_1\|}\,\frac{Z^\ddagger}{Z_0}\,\mathrm{e}^{-\beta V^\ddagger} \\ \chi_{\mathrm{cl}} &= \boldsymbol{\alpha}^{\mathrm{T}}(\beta\widetilde{\mathbb{H}})^{-1}\boldsymbol{\alpha}, \end{aligned} \tag{SI.14}$$

where $V^\ddagger$ is the potential at the MECI relative to the potential of the reactant minimum, $\boldsymbol{G}_0$ and $\boldsymbol{G}_1$ are the gradients on the two potentials and $\widetilde{\mathbb{H}} = (1-\lambda)\mathbb{H}_0 + \lambda\mathbb{H}_1$ is the mixed Hessian given in terms of the Hessians $\mathbb{H}_0$ and $\mathbb{H}_1$, all evaluated at the MECI. The vibrational contributions to the partition functions $Z_0$ and $Z^\ddagger$ are the classical harmonic-oscillator partition functions of the reactant and MECI, where the latter can be evaluated by computing the frequencies of the mixed Hessian $\widetilde{\mathbb{H}}$ after projecting out the reaction

coordinate with $\mathbb{P} = \mathbb{I} - \Delta\boldsymbol{G} \otimes \Delta\boldsymbol{G}^{\mathrm{T}}$. Here $\mathbb{I}$ is the $f$-dimensional identity matrix and $\Delta\boldsymbol{G} = (\boldsymbol{G}_0 - \boldsymbol{G}_1)/\|\boldsymbol{G}_0 - \boldsymbol{G}_1\|$ is the normalized gradient-difference vector.

For globally harmonic model systems such as in Eq. (SI.1), the classical rate [Eq. (SI.14)] simplifies to

$$\begin{aligned} k_{\mathrm{cl}} &= \chi_{\mathrm{MT}}\, f_{\mathrm{FC,MT}}, \\ f_{\mathrm{FC,MT}} &= \frac{1}{\hbar}\sqrt{\frac{\pi\beta}{\Lambda}}\, \mathrm{e}^{-\beta(\Lambda-\varepsilon)^2/4\Lambda}, \\ \chi_{\mathrm{MT}} &= \frac{\alpha^2}{\beta m \omega_{\mathrm{c}}^2}, \end{aligned} \tag{SI.15}$$

where the Marcus-theory Franck–Condon factors $f_{\mathrm{FC,MT}}$ are given in terms of the reorganization energy, $\Lambda = \sum_{i=1}^{f} 2m\Omega_i^2\xi_i^2 = 2m\omega_{\mathrm{t}}^2\zeta_{\mathrm{t}}^2$, and the thermodynamic driving force. In this way the standard Marcus-theory rate (for constant non-zero $\Delta$) given by $k_{\mathrm{MT}} = \Delta^2 f_{\mathrm{FC,MT}}$ is generalized to describe the effect of the CI using the prefactor $\chi_{\mathrm{MT}}$.[16,17]

## SI.2.2 Instanton rates

### SI.2.2.1 Semiclassical instanton theory

The SCI rate for a reaction proceeding via a CI is derived as a semiclassical approximation to Eq. (SI.10). We evaluate the trace in the position representation, insert a complete set of position states in the middle and replace the resulting quantum propagators by semiclassical van-Vleck propagators, which leads to

$$c_{\mathrm{GR}}(\tau + \mathrm{i}t) \sim \frac{1}{\hbar^2}\frac{\sqrt{C_0 C_1}}{(2\pi\hbar)^f} \iint (\boldsymbol{\alpha}\cdot\boldsymbol{x}')(\boldsymbol{\alpha}\cdot\boldsymbol{x}'')\, \mathrm{e}^{-S(\boldsymbol{x}',\boldsymbol{x}'',\tau+\mathrm{i}t)/\hbar}\, \mathrm{d}\boldsymbol{x}'\mathrm{d}\boldsymbol{x}'', \tag{SI.16}$$

where the prefactors, $C_n$, are assumed to vary slowly compared to the exponential and can thus be pulled outside of the integral. In order to carry out these integrals by steepest descent, we locate the stationary point of $S$ (called the instanton) in the combined space of positions and imaginary time and keep $t = 0$ since this is the dominant point of the

correlation function. The action can then be expanded in a Taylor series to second order around the stationary point, which gives

$$c_{\mathrm{GR}}(\bar{\tau}) \sim \frac{1}{\hbar^2}\frac{\sqrt{C_0 C_1}}{(2\pi\hbar)^f}\,\mathrm{e}^{-S(\bar{\boldsymbol{x}}',\bar{\boldsymbol{x}}'',\bar{\tau})/\hbar}\iint(\boldsymbol{\alpha}\cdot\boldsymbol{x}')(\boldsymbol{\alpha}\cdot\boldsymbol{x}'')\,\mathrm{e}^{-(\mathsf{x}-\bar{\mathsf{x}})^{\mathrm{T}}\frac{\partial^2 S}{\partial\mathsf{x}\partial\mathsf{x}}(\mathsf{x}-\bar{\mathsf{x}})/2\hbar}\,\mathrm{d}\boldsymbol{x}'\mathrm{d}\boldsymbol{x}'', \tag{SI.17}$$

where $\mathsf{x} = (\boldsymbol{x}', \boldsymbol{x}'')$ is a vector combining all $2f$ position coordinates and $\frac{\partial^2 S}{\partial\mathsf{x}\partial\mathsf{x}}$ is the second-derivative matrix of the action with respect to these coordinates evaluated at the stationary point with positions $\bar{\mathsf{x}} = (\bar{\boldsymbol{x}}', \bar{\boldsymbol{x}}'')$ and imaginary time $\bar{\tau}$. What remains is a standard multi-dimensional Gaussian integral with monomial prefactors, which evaluates to

$$c_{\mathrm{GR}}(\bar{\tau}) \sim \frac{\chi}{\hbar^2}\sqrt{\frac{C_0 C_1}{C}}\,\mathrm{e}^{-S(\bar{\boldsymbol{x}}',\bar{\boldsymbol{x}}'',\bar{\tau})/\hbar}, \tag{SI.18}$$

where $C$ is the determinant of the second-derivative matrix of the action, $\mathbb{C} = \frac{\partial^2 S}{\partial\mathsf{x}\partial\mathsf{x}}$. The two-point correlation $\chi$ arises from the monomial prefactors inside the integral and is given by $\chi = \hbar\,(\boldsymbol{\alpha}')^{\mathrm{T}}\,\mathbb{C}^{-1}\boldsymbol{\alpha}''$, where $\boldsymbol{\alpha}' = (\boldsymbol{\alpha}, \mathbf{0})$ and $\boldsymbol{\alpha}'' = (\mathbf{0}, \boldsymbol{\alpha})$ are vectors in the space of end points $(\boldsymbol{x}', \boldsymbol{x}'')$ and $\mathbf{0}$ is an $f$-dimensional vector of zeros. The remaining time integral over the correlation function can also be evaluated by steepest descent, which is equivalent to the SCI approach without a CI and again assumes the prefactors, this time including $\chi$, to vary slowly in time compared to $\mathrm{e}^{-S/\hbar}$.[13] The full SCI rate constant is then given by

$$k_{\mathrm{SCI}} = \chi\, f_{\mathrm{FC}},$$
$$f_{\mathrm{FC}} = \frac{\sqrt{2\pi\hbar}}{\hbar^2 Z_0}\sqrt{\frac{C_0 C_1}{C}}\left(-\ddot{S}\right)^{-1/2}\mathrm{e}^{-S(\bar{\boldsymbol{x}}',\bar{\boldsymbol{x}}'',\bar{\tau})/\hbar}, \tag{SI.19}$$

where we used the Cauchy–Riemann equations $\frac{\mathrm{d}^2 S}{\mathrm{d}\tau^2} = -\frac{\mathrm{d}^2 S}{\mathrm{d}t^2}$ and defined $\ddot{S} = \frac{\mathrm{d}^2 S}{\mathrm{d}\tau^2}$, which is negative at the instanton.

As shown in Ref. 15, this derivation can equally be carried out in the ring-polymer formalism and is easily amended to account for rotational and translational contributions. In this ring-polymer version of the instanton rate, presented in Eq. (3.3) of Ref. 15, $\chi$

corresponds to the $(r_{0,\mathrm{c}}, r_{N_\mathrm{R},\mathrm{c}})$-element of the inverse of the second-derivative matrix of the action [defined in Eq. (3.2) of Ref. 15] with respect to all bead positions multiplied by $\hbar\alpha^2$. Note that in Ref. 15, the reactant and product states were labelled by $n \in \{\mathrm{R}, \mathrm{P}\}$, whereas in this work we use $n \in \{0, 1\}$. The notation of Ref. 15 uses the notation $r_{0,\mathrm{c}}, r_{N_\mathrm{R},\mathrm{c}}$ to refer to the coupling coordinate of the hopping beads, $x_\mathrm{c}$, with $i = 0$ and $i = N_\mathrm{R}$.

#### SI.2.2.2 Steepest-descent instanton theory

The calculation of the SDI rate is conceptually similar to the calculation of the standard golden-rule instanton rate without CI with the exception that the diabatic coupling is included in the action [Eq. (4) of the main text], which may lead to the occurrence of several stationary paths of the effective action, $S^\mathrm{eff} = S - \hbar \ln|\Delta(\boldsymbol{x}')\Delta(\boldsymbol{x}'')|$. This is illustrated in Fig. SI.2, where we plot the integrand of Eq. (SI.16) for the three-mode model defined in Sec. SI.1 in the $x_\mathrm{c}'x_\mathrm{c}''$ coordinate space. From the figure, we can identify two positive lobes, corresponding to the direct paths that bypass the CI and contribute to the total rate with a positive sign, as well as two negative lobes, corresponding to the winding pathways that give rise to a negative contribution to the rate (see Fig. SI.5 and Fig. 1 of the main text for the pathways). Note that the reason for the negative contribution associated with the winding paths is that they employ two different hopping points, $\bar{\boldsymbol{x}}' \neq \bar{\boldsymbol{x}}''$, on opposite sides of the CI.

The total rate is thus given by a sum over the steepest-descent rates associated with each of these stationary paths

$$\begin{aligned} k_\mathrm{SDI} &= \sum_a \chi_a^\mathrm{eff} f_{\mathrm{FC},a}^\mathrm{eff}, \\ f_{\mathrm{FC},a}^\mathrm{eff} &= \frac{\sqrt{2\pi\hbar}}{\hbar^2 Z_0} \sqrt{\frac{C_0\, C_1}{C^\mathrm{eff}}} \left(-\frac{\mathrm{d}^2 S^\mathrm{eff}}{\mathrm{d}\tau^2}\right)^{-1/2} \mathrm{e}^{-S^\mathrm{eff}(\bar{\boldsymbol{x}}_a', \bar{\boldsymbol{x}}_a'', \bar{\tau}_a)/\hbar}, \end{aligned} \tag{SI.20}$$

where all quantities are evaluated for the respective stationary path of $S^\mathrm{eff}$. Here, the $C^\mathrm{eff}$ prefactor is defined in analogy to $C$ in Eq. (SI.18) but with respect to the effective action in-

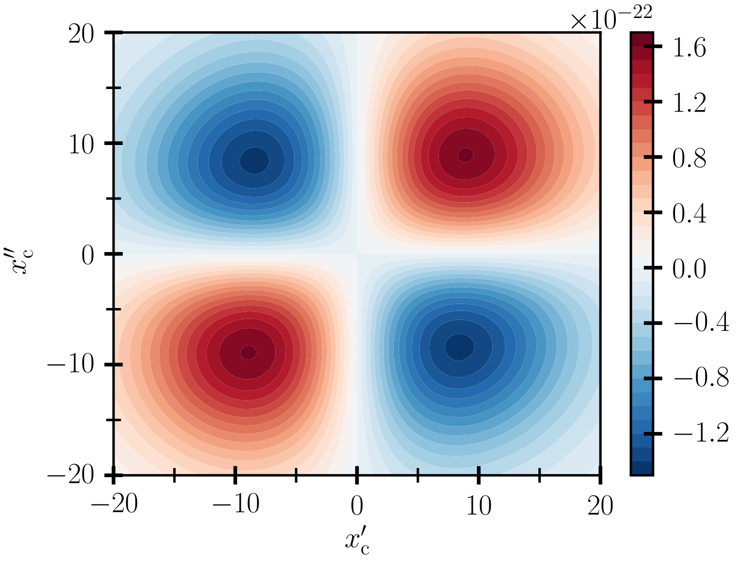


Figure SI.2: Colour map of the integrand in Eq. (SI.16) for the model system defined in Sec SI.1 in atomic units. The two positive lobes correspond to the direct paths in the SDI formalism, while the two negative lobes correspond to the winding paths (see also Fig. SI.5). To construct the plot, we transformed the system to a spin-boson model, as described in Eq. (SI.6), and used the corresponding analytic expressions for the action.[13] The two remaining coordinates, as well as the value of $\tau$ were fixed to their values at the stationary point of the (effective) action. Note that in this symmetric spin-boson model, the stationary points in the SCI and SDI formalisms only differ in the coupling coordinate, while the values of the two other modes and $\tau$ are the same.

stead of $S$. We thereby separated the range of the position integrals into different regions, $\Gamma_a$ around the stationary points (similarly to Ref. 18). The prefactors, $\chi_a^{\text{eff}} = \text{sgn}[\Delta(\bar{\boldsymbol{x}}')\Delta(\bar{\boldsymbol{x}}'')]$, depend on the relative sign of the diabatic-coupling terms at the hopping points of the stationary paths. In particular, direct instantons for which $\bar{\boldsymbol{x}}' = \bar{\boldsymbol{x}}''$ have $\chi_a^{\text{eff}} = 1$, whereas those which wind around the CI have $\chi_a^{\text{eff}} = -1$. The derivatives of the coupling also enter into the second-derivative matrix $C^{\text{eff}}$ and the total $\tau$-derivative since these terms derive from $S^{\text{eff}}$.

In Table 1 of the main text, we presented the SDI rate constant for the three-mode model defined above. Note that the 20% deviation from the quantum-mechanical result arises from the neglect of anharmonicity around the stationary points of $S_{\text{eff}}$, which within SDI is not quadratic even for the harmonic LVC model, because of the contribution from the diabatic coupling.

### SI.2.3 Resummed semiclassical instanton theory

We presented the SCI result for the three-mode model in Table 1 of the main text, which deviates from the FGR rate constant by a factor of around 2. Considering all error sources in realistic applications, including the electronic-structure calculations, this still constitutes decent agreement. However, we can understand the reason for the deviation by comparing the flux correlation functions underlying the different methods, which are illustrated in Fig. SI.3(a) at the stationary value of $\tau = \beta\hbar/2$.

In previous instanton studies on reactions without CIs,[19] the GR flux correlation function at the stationary value of $\tau$ was shown to be almost perfectly described by a Gaussian. This is not the case in Fig. SI.3(a), which exhibits dips to negative functional values. This is caused by the two-point correlation, $\chi(\tau + \mathrm{i}t)$, which fluctuates relatively quickly in time. The SCI approximation of the correlation function treats $\chi$ as a constant and thus overestimates the rate. The SDI approach captures the negative contributions to the correlation function by virtue of locating several instantons with positive and negative contributions to the rate, see also Fig. SI.3(b). This leads to the improved agreement with the GR rate observed in Table SI.2.

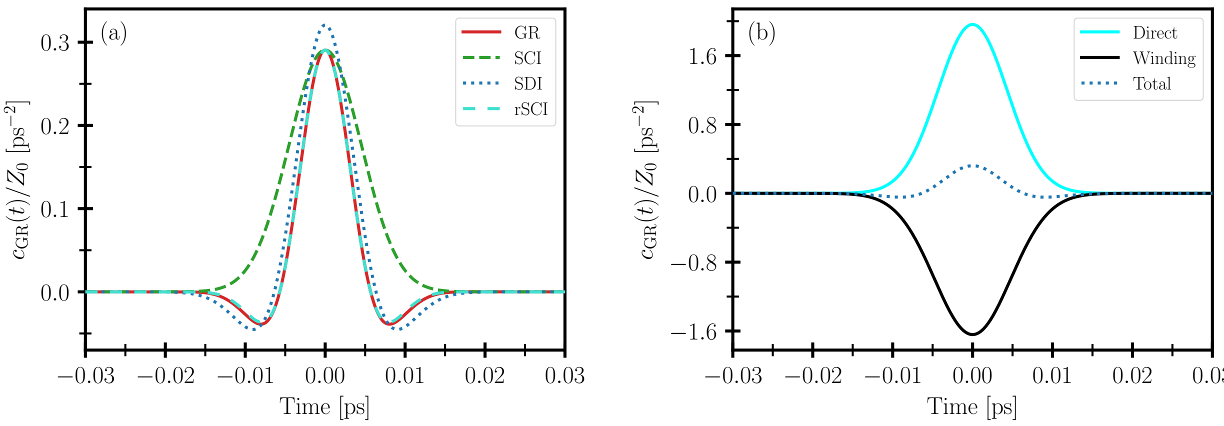


Figure SI.3: (a) Flux correlation function of the three-mode model defined in the text computed with the quantum-mechanical GR, steepest-descent (SDI) and semiclassical instanton theory (SCI), as well as with resummed semiclassical instanton theory (rSCI). In all cases, we set $\tau$ to its value at the stationary point. (b) Contributions to the total flux correlation function in SDI from the direct and winding instantons.

Based on our understanding of the error in SCI, we can improve the method by at least partially correcting for a rapidly oscillating prefactor. First, we go beyond the constant approximation for $\chi$ by expanding it as a second-order Taylor series. Next, we can use our knowledge that the analytical expression for $\chi$ in a harmonic system [Eqs. (SI.12a)] is a cosh function. Given $\chi(\bar{\tau})$ and its second $\tau$-derivative we can therefore propose a resummation for the Taylor series as

$$\chi(\bar{\tau}+\mathrm{i}t) = \chi(\bar{\tau}) \left[1 + \frac{1}{2}\frac{\ddot{\chi}(\bar{\tau})}{\chi(\bar{\tau})}(\mathrm{i}t)^2 + \cdots\right] \sim \chi(\bar{\tau}) \cos\left(\sqrt{\frac{\ddot{\chi}(\bar{\tau})}{\chi(\bar{\tau})}}\, t\right), \tag{SI.21}$$

where $\ddot{\chi} = \frac{\partial^2 \chi}{\partial \tau^2}$, and we used the relation $\cosh(\mathrm{i}t) = \cos(t)$. The corresponding approximation of the correlation function, depicted in Fig. SI.3(a), is in excellent agreement with the quantum-mechanical GR correlation function. With knowledge of $\chi(\tau)$, we can approximate the exponential in Eq. (SI.18) by a Gaussian in the time domain and take the final integral over time analytically.

In order to carry out this approach for an anharmonic system, we need to compute the second time-derivative of $\chi$ at $\tau = \bar{\tau}$. This can be achieved by taking time-derivatives of the correlation function, as can be seen from Eq. (SI.18). Taking the second time-derivative of the golden-rule flux correlation function [Eq. (SI.10)] leads to

$$\begin{aligned}\ddot{c}_{\mathrm{GR}}(\tau+\mathrm{i}t) = \frac{\partial^2 c_{\mathrm{GR}}(\tau+\mathrm{i}t)}{\partial \tau^2} = \frac{1}{m^2\hbar^2} \operatorname{tr}\left\{(\boldsymbol{\alpha}\cdot\hat{\boldsymbol{p}})\, \mathrm{e}^{-\hat{H}_0(\beta\hbar-\tau-\mathrm{i}t)/\hbar}\, (\boldsymbol{\alpha}\cdot\hat{\boldsymbol{p}})\, \mathrm{e}^{-\hat{H}_1(\tau+\mathrm{i}t)/\hbar}\right\} \\ + \frac{1}{\hbar^4} \operatorname{tr}\left\{[V_0(\hat{\boldsymbol{x}}) - V_1(\hat{\boldsymbol{x}})](\boldsymbol{\alpha}\cdot\hat{\boldsymbol{x}})\, \mathrm{e}^{-\hat{H}_0(\beta\hbar-\tau-\mathrm{i}t)/\hbar}\, [V_0(\hat{\boldsymbol{x}}) - V_1(\hat{\boldsymbol{x}})](\boldsymbol{\alpha}\cdot\hat{\boldsymbol{x}})\, \mathrm{e}^{-\hat{H}_1(\tau+\mathrm{i}t)/\hbar}\right\},\end{aligned} \tag{SI.22}$$

where the second term on the right-hand side arises from time-derivatives of the time-evolution operators and is thus also found in Wolynes theory,[6,20,21] even in cases where the position-dependence of the diabatic coupling is ignored. It follows that the first term on the right-hand side of Eq. (SI.22), which contains time-derivatives of the diabatic coupling,

contains the information about $\ddot{\chi}$. In particular, the term of interest is given by

$$\frac{\ddot{\chi}(\tau + \mathrm{i}t)}{\chi(\tau + \mathrm{i}t)} = \frac{1}{m^2} \frac{\mathrm{tr}\left\{(\boldsymbol{\alpha} \cdot \hat{\boldsymbol{p}})\, \mathrm{e}^{-\hat{H}_0(\beta\hbar - \tau - \mathrm{i}t)/\hbar}\, (\boldsymbol{\alpha} \cdot \hat{\boldsymbol{p}})\, \mathrm{e}^{-\hat{H}_1(\tau + \mathrm{i}t)/\hbar}\right\}}{\mathrm{tr}\left\{(\boldsymbol{\alpha} \cdot \hat{\boldsymbol{x}})\, \mathrm{e}^{-\hat{H}_0(\beta\hbar - \tau - \mathrm{i}t)/\hbar}\, (\boldsymbol{\alpha} \cdot \hat{\boldsymbol{x}})\, \mathrm{e}^{-\hat{H}_1(\tau + \mathrm{i}t)/\hbar}\right\}}. \tag{SI.23}$$

As before, we expand this expression in a position basis, replace the quantum propagators by their semiclassical analogues and evaluate the term around the instanton (the stationary point of the total action). At the instanton, the component of the momentum pointing along the coupling mode (orthogonal to the reaction coordinate given by the tuning mode) is zero. We therefore need to use the following expansion

$$\begin{aligned} \langle \boldsymbol{x}' | (\boldsymbol{\alpha} \cdot \hat{\boldsymbol{p}})\, \mathrm{e}^{-\hat{H}_n \tau_n/\hbar} | \boldsymbol{x}'' \rangle &\sim -\mathrm{i}\hbar \left( \boldsymbol{\alpha} \cdot \frac{\partial}{\partial \boldsymbol{x}'} \right) \sqrt{\frac{C_n}{(2\pi\hbar)^f}}\, \mathrm{e}^{-S_n/\hbar} \\ &\sim \mathrm{i}\boldsymbol{\alpha} \cdot \left( \frac{\partial^2 S_n}{\partial \boldsymbol{x}' \partial \boldsymbol{x}'}\, \boldsymbol{x}' + \frac{\partial^2 S_n}{\partial \boldsymbol{x}' \partial \boldsymbol{x}''}\, \boldsymbol{x}'' \right) \sqrt{\frac{C_n}{(2\pi\hbar)^f}}\, \mathrm{e}^{-S_n/\hbar}, \end{aligned} \tag{SI.24}$$

where all derivatives are evaluated at the hopping points. The analogous procedure is followed for the terms associated with the propagation in the other direction. Second derivatives of $S_n$ can be evaluated using formulas presented in the Appendix of Ref. 22. These require knowledge of nothing more than the potentials, gradients and Hessians along the instanton path, which are anyway required for the original SCI approach.

We are now in the position to evaluate $\ddot{\chi}$ at the instanton, which is given by

$$\ddot{\chi}(\bar{\tau}) = -\frac{\hbar}{m^2} (\boldsymbol{\alpha}')^{\mathrm{T}} \begin{pmatrix} \frac{\partial^2 S_0}{\partial \boldsymbol{x}' \partial \boldsymbol{x}'} & \frac{\partial^2 S_0}{\partial \boldsymbol{x}' \partial \boldsymbol{x}''} \\ \frac{\partial^2 S_0}{\partial \boldsymbol{x}'' \partial \boldsymbol{x}'} & \frac{\partial^2 S_0}{\partial \boldsymbol{x}'' \partial \boldsymbol{x}''} \end{pmatrix} \begin{pmatrix} \frac{\partial^2 S}{\partial \boldsymbol{x}' \partial \boldsymbol{x}'} & \frac{\partial^2 S}{\partial \boldsymbol{x}' \partial \boldsymbol{x}''} \\ \frac{\partial^2 S}{\partial \boldsymbol{x}'' \partial \boldsymbol{x}'} & \frac{\partial^2 S}{\partial \boldsymbol{x}'' \partial \boldsymbol{x}''} \end{pmatrix}^{-1} \begin{pmatrix} \frac{\partial^2 S_1}{\partial \boldsymbol{x}' \partial \boldsymbol{x}'} & \frac{\partial^2 S_1}{\partial \boldsymbol{x}' \partial \boldsymbol{x}''} \\ \frac{\partial^2 S_1}{\partial \boldsymbol{x}'' \partial \boldsymbol{x}'} & \frac{\partial^2 S_1}{\partial \boldsymbol{x}'' \partial \boldsymbol{x}''} \end{pmatrix} \boldsymbol{\alpha}'', \tag{SI.25}$$

and the masses are accounted for by mass-weighting all terms. With the general expressions

for $\chi(\bar{\tau})$ and $\ddot{\chi}(\bar{\tau})$ at hand, the resummed SCI formula for the rate constant is given by

$$\begin{aligned} k_{\mathrm{rSCI}} &= \frac{1}{\hbar^2 Z_0}\sqrt{\frac{C_0 C_1}{C}}\,\mathrm{e}^{-S(\bar{\tau})/\hbar}\int_{-\infty}^{\infty}\chi(\bar{\tau}+\mathrm{i}t)\,\mathrm{e}^{\frac{t^2}{2\hbar}\ddot{S}(\bar{\tau})}\,\mathrm{d}t, \\ &= \chi_{\mathrm{r}}\,f_{\mathrm{FC}}, \end{aligned} \tag{SI.26}$$

where $\chi_{\mathrm{r}} = \chi\,\mathrm{e}^{\frac{\hbar}{2}\frac{\ddot{\chi}}{\chi}\ddot{S}^{-1}}$, and all quantities are evaluated at the instanton. We present the rate constant for the three-mode model obtained with this method in Table SI.2. It is seen to be in excellent agreement with the quantum-mechanical results, as expected for the harmonic system. For more complicated, anharmonic systems where the diagonal and off-diagonal blocks of the $\mathbb{C}$-matrix cannot be simultaneously diagonalized, the resummation would have to be adapted. We leave the development of such a more general resummation open for future work since the correction of the SCI rate in the model system is relatively minor.

### SI.2.4 Extended results for the model system

In Table SI.2, we compare the classical and NA-TST rates to the other rates given in Table 1 of the main text. Furthermore, we illustrate the rate constants computed with several methods over a range of different temperatures in Fig. SI.4. As also seen in our *ab initio* results, the NA-TST rate for the model system can be larger than the instanton and quantum-mechanical rates, despite the neglect of nuclear tunnelling in this method. Furthermore, Table SI.2 seems to suggest that the classical rate is in reasonably good agreement with the exact result. This is simply a fortuitous cancellation of errors, on which one cannot rely in general.

Table SI.2: Reaction rates [in $\mathrm{ns}^{-1}$] for 300 K from Table 1 of the main text in addition to the classical and NA-TST rates.

| BOI | Classical | NA-TST | SCI | SDI | rSCI | GR | Exact |
|---|---|---|---|---|---|---|---|
| 2401 | 0.9706 | 30.95 | 3.306 | 1.784 | 1.507 | 1.473 | 1.475 |

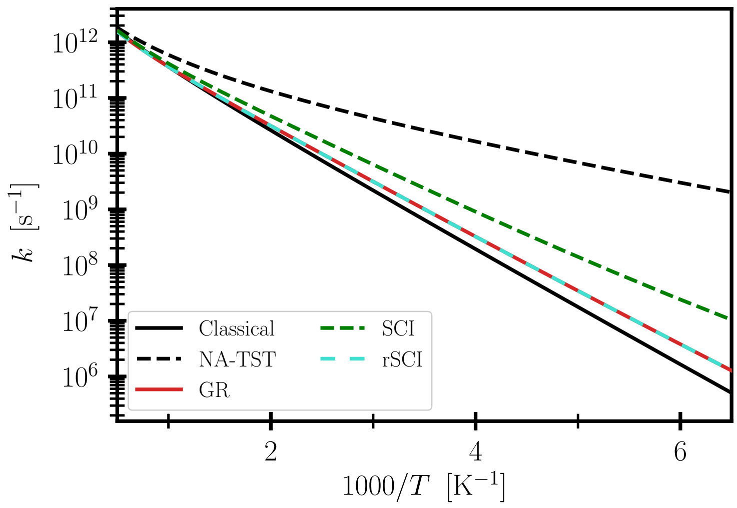


Figure SI.4: Rates computed with different methods for the three-mode model system.

# SI.3 SDI and the geometric phase

It is also possible to define a classical rate in the spirit of the SDI approach. Therefore, we evaluate the correlation function [Eq. (SI.7)] by a phase-space integral but this time include the coupling in the exponent. We then arrive at a configuration-space integral that we can take by steepest descent about the stationary points of the effective potential $V^{\text{eff}}(\boldsymbol{x}, \lambda) = (1-\lambda)V_0(\boldsymbol{x}) + \lambda V_1(\boldsymbol{x}) - \ln[\Delta(\boldsymbol{x})^2]/\beta$. This leads to the rate expression

$$
\begin{aligned}
k_{\text{sdcl}} &= \sum_a \chi_{\text{cl}}^{\text{eff}} f_{\text{FC,cl},a}^{\text{eff}}, \\
f_{\text{FC,cl},a}^{\text{eff}} &= \sqrt{\frac{2\pi m}{\beta}} \frac{1}{\hbar^2 \|\boldsymbol{G}_{0,a} - \boldsymbol{G}_{1,a}\|} \frac{Z_a^{\text{eff},\ddagger}}{Z_0} \, \mathrm{e}^{-\beta V_a^{\text{eff},\ddagger}},
\end{aligned}
\tag{SI.27}
$$

where the sum is over stationary points of the effective potential, the MECI partition function, $Z^{\text{eff},\ddagger}$, is evaluated based on the second-derivative matrix of $V^{\text{eff}}$ with respect to positions only with the reaction coordinate projected out. Finally, $\chi_{\text{cl}}^{\text{eff}} = 1$ is the classical limit of $\chi^{\text{eff}}$.

For the model system from Sec. SI.1, the method leads to a rate of $1.010\,\text{ns}^{-1}$, which is in excellent agreement with the classical rate in Table SI.2 obtained with Eq. (SI.14). Note that in the effective SDI action [Eq. (4)], $\Delta(\boldsymbol{x}')$ and $\Delta(\boldsymbol{x}'')$ can vary independently giving

rise to two pairs of stationary paths, one pair with a positive sign and the other pair with a negative sign. In contrast, the $\Delta(\boldsymbol{x})^2$ term in the effective potential of Eqs. (SI.27) only allows for one pair of stationary paths with a positive sign. The classical rate therefore does not contain negative contributions and cannot account for the GPE. The lack of GPEs in the classical and NA-TST rates is the reason why these rates can be higher than the instanton and quantum-mechanical results, as shown in Fig. SI.4, despite the neglect of tunnelling.

The SDI instantons are depicted in Fig. SI.5 along with the stationary points of $V^{\text{eff}}$ projected onto the two-dimensional plane spanned by $x_{\text{t}}$ and $x_{\text{c}}$. The Figure confirms that the two stationary points of the effective potential correspond to the classical limit of the curving or *direct* instantons (blue and red paths), whereas the pair of instantons *winding* around the CI (violet paths, on top of each other, winding clockwise and counterclockwise), which reduce the total rate, do not have a classical analogue.

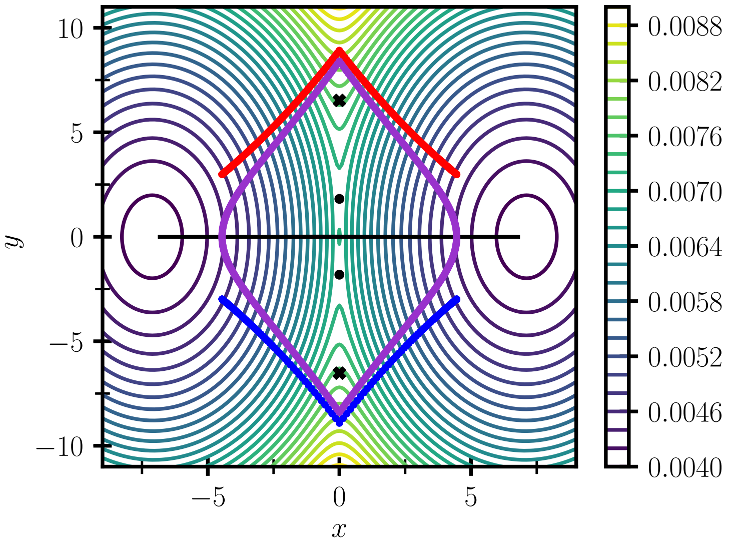


Figure SI.5: Contour plot of the adiabatic ground-state potential projected into the $x_{\text{t}}x_{\text{c}}$-plane (with a relaxed bath-mode coordinate) along with the steepest-descent instantons and the stationary points of $V^{\text{eff}}$ (black crosses) defined beneath Eq. (SI.27) at 300 K. The *direct* blue and red paths contribute to the rate with a positive sign, whereas the *winding* violet paths (clockwise and counterclockwise, lie on top of each other) contribute with a negative sign. Additionally, the Born–Oppenheimer instantons (black lines) and transition states (black dots) are shown. Note that there are two BOI paths curving left and right around the CI, which are not resolved here because the paths traverse the barrier very close to the CI.

It can further be seen that the curving SDI paths deviate significantly from the corresponding classical stationary points. In both the instanton approach and the classical approach, a compromise needs to be found between maximizing the diabatic coupling, which grows away from the CI, and minimizing the potential-energy barrier, which is smallest at the CI. Such effects of corner cutting, where tunnelling changes the reaction mechanism, are not uncommon. Usually, however, the instanton cuts the corner to find a shorter, more direct path from reactant to product at the expense of travelling through regions of higher potential energy. This can be more favourable because tunnelling through high but narrow barriers is very efficient. In this case, the steepest-descent instantons take a longer path exhibiting a higher barrier than the path via the classical stationary points, which is favourable because of the larger diabatic coupling along these paths. The ability of the instanton to describe tunnelling hence allows it to find a better (in fact the optimal) compromise between path length, potential-energy barrier and diabatic coupling than classical rate theory.

## SI.4 Ab initio calculations

In this section we present additional details of the *ab initio* calculations and additional discussions on the *ab initio* results.

### SI.4.1 PBE vs PBEX50 functional

Hybrid functionals, often with an increased fraction of Fock exchange, are commonly used in constrained density functional theory (CDFT) calculations, especially for computing diabatic coupling.[23,24] Since the PBE functional is considerably less expensive than the PBEX50 functional (i.e. a hybrid functional with 50% Fock exchange), we employed it for the instanton optimizations. We have carefully tested its performance compared to the PBEX50 functional. The fully optimized geometries with the PBEX50 functional are almost identical to those of the PBE functional, and the MECP barrier is 0.180 eV (only 0.009 eV higher than the PBE

barrier). Therefore we think that it is justifiable to use the PBE functional in the geometry (including instanton) optimizations.

### SI.4.2 Coupling mode from CDFT

Since the coupling mode vector is defined as $\boldsymbol{\alpha} = \frac{\partial \Delta}{\partial \boldsymbol{x}}$, it seems one could straightforwardly perform a finite displacement calculation at the MECI to obtain $\boldsymbol{\alpha}$. However, CDFT is only able to return unsigned $|\Delta|$ values, making the procedure to obtain $\boldsymbol{\alpha}$ more complicated as $|\Delta|$ is not a differentiable function at the CI. We first perform forward-difference and backward-difference calculations, and by taking the unsigned average of the two (i.e., the forward-difference derivative has only positive elements and the backward-difference derivative has only negative elements so we reverse the backward-difference derivative before averaging), we obtain $\widetilde{\boldsymbol{\alpha}} = \frac{\partial |\Delta|}{\partial \boldsymbol{x}}$. This vector has the correct magnitude (i.e. the same as $\boldsymbol{\alpha}$), but the relative signs for each element are missing. Next we displace the MECI in an arbitrary direction by a displacement larger than the step size used for the numerical derivative. At this point, we perform a central-difference gradient calculation to obtain the signs of the elements of $\boldsymbol{\alpha}$. The procedure is repeated to ensure consistency by recomputing the gradient at a point displaced from the CI in the direction of $\boldsymbol{\alpha}$, where the derivative of $|\Delta|$ is well defined. Finally, we checked that displacing along $\boldsymbol{\alpha}$ does not lift the degeneracy of the two diabatic states and that the slope of $|\Delta|$ along this mode is indeed $\alpha = ||\boldsymbol{\alpha}||$.

### SI.4.3 Rate constants and H/D kinetic isotope effects

Here we present all the computed rate constants for BMA and deuterated BMA. One can see from Table SI.3 (comparing the top and bottom sections) that the H/D kinetic isotope effect (KIE) predicted by NA-TST is 1.2 at 300 K, while the KIE predicted by SCI is 1.4. At 150 K, KIE only slightly increased (to 2.3) according to SCI, only slightly larger than the NA-TST KIE (1.4). By comparing the decompositions in Table SI.4 and Table 2 in the main text, one can see that apart from the ZPE contributions to the H/D isotope effect

(which appear in the NA-TST KIEs), the remainder mainly originates from the tunnelling factor. Meanwhile, if one substitutes all $^{12}$C atoms with $^{13}$C, the tunnelling factor decreases from 9.1 to 8.6 at 300 K, which as discussed in the main text, is a remarkably large KIE for heavy-atom substitution at room temperature.

Table SI.3: Rate constants (in units of $ns^{-1}$) for the charge transfer process in BMA cation (top) and deuterated BMA cation (bottom) computed with CDFT.

| $T$ [K] | $k_{cl}$ | $k_{NA\text{-}TST}$ | $k_{SCI}$ |
|---|---|---|---|
| 370 | 24 | 90 | 81 |
| 300 | 6.3 | 39 | 45 |
| 250 | 1.5 | 17 | 24 |
| 200 | 0.19 | 5.1 | 11 |
| 150 | 0.0061 | 0.74 | 6.3 |
| 300 | 6.2 | 33 | 32 |
| 200 | 0.19 | 3.8 | 7.1 |
| 150 | 0.0060 | 0.52 | 2.7 |

Table SI.4: Same as Table 2 in the main text but for fully deuterated BMA.

| $T$ [K] | ZPE | Tunnelling | CI | Fluctuations |
|---|---|---|---|---|
| 300 | 5.2 | 7.1 | 0.56 | 0.24 |
| 200 | 21 | 66 | 0.38 | 0.073 |
| 150 | 87 | 830 | 0.29 | 0.022 |

Table SI.5: The contribution from fluctuations is defined as the ratio between SCI and NA-TST prefactors computed on the full-dimensional system vs 1D MEP.

| $T$ [K] | full dimensional | 1D MEP |
|---|---|---|
| 370 | 0.38 | 0.59 |
| 300 | 0.26 | 0.51 |
| 250 | 0.15 | 0.45 |
| 200 | 0.069 | 0.41 |
| 150 | 0.023 | 0.40 |

### SI.4.4 1D vs full-dimensional representation and discussion on the fluctuations term

Given the seemingly negligible corner-cutting effects, one might expect that a one-dimensional description based on the minimum-energy pathways (MEPs) would be sufficient. However, the contribution from fluctuations (i.e. the ratio between SCI and NA-TST prefactor) at 150 K would be 0.40 if computed on the one-dimensional MEPs, significantly larger than the value (0.023) computed on the full-dimensional system. One can see from Table SI.5 that the contribution from fluctuations decreases rapidly with temperature for the full-dimensional system, while it changes much slower for the 1D MEPs. This indicates that a theory based on the one-dimensional MEP is inadequate to describe the system, and that the fluctuations around the instanton contain important multidimensional effects. A similar behaviour of the fluctuations has also been found for the LVC model (see Table SI.6). This indicates that this effect is not unique to the *ab initio* system and that it can be reproduced with multidimensional globally harmonic models.

## SI.5 Ab initio system vs LVC model

In Fig. SI.6, we compare our first-principles results based on CDFT with rate constants computed for the full-dimensional LVC model constructed in Ref. 25. It can be seen that the SCI result for the LVC model at 300 K is in agreement with the GR rate constant extracted from the short-time dynamics shown in Fig. 18 of Ref. 25. This is further evidence that the semiclassical approximation inherent in instanton theory is well-suited for the description of the charge transfer in the BMA cation. While the first-principles results agree with the rate constants for the LVC model at high temperatures, a significant deviation of more than one order of magnitude is observed in Fig. SI.6 at lower temperatures. One reason for this difference is that different electronic-structure methods were used in the two studies. In particular, CASSCF was used to construct the LVC model, whereas our first-principles calculations are

Table SI.6: Same as Table 2 in the main text but for the LVC model.

| T | ZPE | Tunnelling | CI | Fluctuations |
|---|---|---|---|---|
| 370 | 4.2 | 15 | 0.53 | 0.46 |
| 300 | 7.4 | 54 | 0.44 | 0.33 |
| 250 | 14 | 230 | 0.38 | 0.22 |
| 200 | 38 | 2200 | 0.34 | 0.090 |
| 150 | 220 | 97000 | 0.36 | 0.014 |

based on CDFT. A second difference is that the LVC model uses a global harmonic approximation, whereas our first-principles instanton calculations make no assumptions about the shape of the underlying PESs.

The comparison between the different contributions to the SCI rates based on our on-the-fly CDFT calculations (Table 2 of the main text) and the LVC model (Table SI.6) highlights that the tunnelling contribution constitutes the decisive difference between the two approaches. This deviation originates from differing instanton actions, indicating that the barrier in the harmonic LVC model is narrower than the one obtained with on-the-fly CDFT.

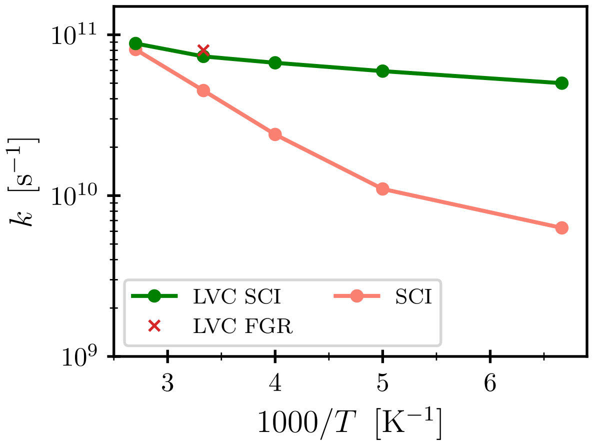


Figure SI.6: Rate constants computed with SCI for the charge transfer in the BMA cation based on on-the-fly CDFT evaluations of the PESs. For comparison, we present results for the full-dimensional LVC model of Ref. 25. The "LVC FGR" results are extracted from Fig. 18 of Ref. 25, and the "LVC SCI" results are computed with instanton theory on the same LVC model.

# References


(1) Ryabinkin, I. G.; Joubert-Doriol, L.; Izmaylov, A. F. When do we need to account for the geometric phase in excited state dynamics? *J. Chem. Phys.* **2014**, *140*, 214116.

(2) Maskri, R.; Joubert-Doriol, L. The moving crude adiabatic alternative to the adiabatic representation in excited state dynamics. *Philos. Trans. R. Soc. A* **2022**, *380*, 20200379.

(3) Yamamoto, T. Quantum Statistical Mechanical Theory of the Rate of Exchange Chemical Reactions in the Gas Phase. *J. Chem. Phys.* **1960**, *33*, 281.

(4) Miller, W. H. Quantum mechanical transition state theory and a new semiclassical model for reaction rate constants. *J. Chem. Phys.* **1974**, *61*, 1823.

(5) Miller, W. H.; Schwartz, S. D.; Tromp, J. W. Quantum mechanical rate constants for bimolecular reactions. *J. Chem. Phys.* **1983**, *79*, 4889–4898.

(6) Wolynes, P. G. Imaginary time path integral Monte Carlo route to rate coefficients for nonadiabatic barrier crossing. *J. Chem. Phys.* **1987**, *87*, 6559–6561.

(7) Chandler, D. In *Classical and Quantum Dynamics in Condensed Phase Simulations*; Berne, B. J., Ciccotti, G., Coker, D. F., Eds.; World Scientific: Singapore, 1998; Chapter 2, pp 25–49.

(8) Dirac, P. A. M. The quantum theory of the emission and absorption of radiation. *Proc. R. Soc. London A.* **1927**, *114*, 243–265.

(9) Wentzel, G. Über strahlungslose Quantensprünge. *Z. Phys.* **1927**, *43*, 524–530.

(10) Zwanzig, R. *Nonequilibrium Statistical Mechanics*; Oxford University Press: New York, 2001.

(11) Nitzan, A. *Chemical Dynamics in Condensed Phases: Relaxation, Transfer, and Reactions in Condensed Molecular Systems*; Oxford University Press: Oxford, 2006.

(12) Feynman, R. P.; Hibbs, A. R. *Quantum Mechanics and Path Integrals*; McGraw-Hill: New York, 1965.

(13) Richardson, J. O.; Bauer, R.; Thoss, M. Semiclassical Green's functions and an instanton formulation of electron-transfer rates in the nonadiabatic limit. *J. Chem. Phys.* **2015**, *143*, 134115.

(14) Koga, N.; Morokuma, K. Determination of the lowest energy point on the crossing seam between two potential surfaces using the energy gradient. *Chem. Phys. Lett.* **1985**, *119*, 371–374.

(15) Ansari, I. M.; Heller, E. R.; Trenins, G.; Richardson, J. O. Instanton theory for Fermi's golden rule and beyond. *Phil. Trans. R. Soc. A.* **2022**, *380*, 20200378.

(16) Daizadeh, I.; Medvedev, E. S.; Stuchebrukhov, A. A. Effect of protein dynamics on biological electron transfer. *Proc. Natl. Acad. Sci.* **1997**, *94*, 3703–3708.

(17) Medvedev, E. S.; Stuchebrukhov, A. A. Inelastic tunneling in long-distance biological electron transfer reactions. *J. Chem. Phys.* **1997**, *107*, 3821–3831.

(18) Jahr, E.; Laude, G.; Richardson, J. O. Instanton theory of tunnelling in molecules with asymmetric isotopic substitutions. *J. Chem. Phys.* **2020**, *153*, 094101.

(19) Heller, E. R.; Richardson, J. O. Instanton formulation of Fermi's golden rule in the Marcus inverted regime. *J. Chem. Phys.* **2020**, *152*, 034106.

(20) Thapa, M. J. Path Integral Methods in Quantum Rate Theories. Ph.D. thesis, ETH Zurich, 2020.

(21) Fang, W.; Thapa, M. J.; Richardson, J. O. Nonadiabatic quantum transition-state theory in the golden-rule limit: II. Overcoming the pitfalls of the saddle-point and semiclassical approximations. *J. Chem. Phys.* **2019**, *151*, 214101.

(22) Richardson, J. O. Ring-polymer instanton theory of electron transfer in the nonadiabatic limit. *J. Chem. Phys.* **2015**, *143*, 134116.

(23) Kubas, A.; Hoffmann, F.; Heck, A.; Oberhofer, H.; Elstner, M.; Blumberger, J. Electronic couplings for molecular charge transfer: Benchmarking CDFT, FODFT, and FODFTB against high-level ab initio calculations. *J. Chem. Phys.* **2014**, *140*, 104105.

(24) Kubas, A.; Gajdos, F.; Heck, A.; Oberhofer, H.; Elstner, M.; Blumberger, J. Electronic couplings for molecular charge transfer: benchmarking CDFT, FODFT and FODFTB against high-level ab initio calculations. II. *Phys. Chem. Chem. Phys.* **2015**, *17*, 14342–14354.

(25) Izmaylov, A. F.; Mendive–Tapia, D.; Bearpark, M. J.; Robb, M. A.; Tully, J. C.; Frisch, M. J. Nonequilibrium Fermi golden rule for electronic transitions through conical intersections. *J. Chem. Phys.* **2011**, *135*, 234106.
